# High-Dimensional Profiling Reveals Heterogeneity of the Th17 Subset and Its Association With Systemic Immunomodulatory Treatment in Non-infectious Uveitis

**DOI:** 10.3389/fimmu.2018.02519

**Published:** 2018-10-31

**Authors:** Fleurieke H. Verhagen, Sanne Hiddingh, Rianne Rijken, Aridaman Pandit, Emmerik Leijten, Michel Olde Nordkamp, Ninette H. ten Dam-van Loon, Stefan Nierkens, Saskia M. Imhof, Joke H. de Boer, Timothy R. D. J. Radstake, Jonas J. W. Kuiper

**Affiliations:** ^1^Ophthalmo-Immunology Unit, University Medical Center Utrecht, Utrecht University, Utrecht, Netherlands; ^2^Department of Ophthalmology, University Medical Center Utrecht, Utrecht University, Utrecht, Netherlands; ^3^Laboratory of Translational Immunology, University Medical Center Utrecht, Utrecht University, Utrecht, Netherlands; ^4^Department of Rheumatology & Clinical Immunology, University Medical Center Utrecht, Utrecht University, Utrecht, Netherlands

**Keywords:** flow cytometry, non-infectious uveitis, immunosuppressive therapy, Th17, CCR6

## Abstract

**Background:** Non-infectious uveitis (NIU) is a severe intra ocular inflammation, which frequently requires prompt systemic immunosuppressive therapy (IMT) to halt the development of vision-threatening complications. IMT is considered when NIU cannot be treated with corticosteroids alone, which is unpredictable in advance. Previous studies have linked blood cell subsets to glucocorticoid sensitivity, which suggests that the composition of blood leukocytes may early identify patients that will require IMT.

**Objective:** To map the blood leukocyte composition of NIU and identify cell subsets that stratify patients that required IMT during follow-up.

**Methods:** We performed controlled flow cytometry experiments measuring a total of 37 protein markers in the blood of 30 IMT free patients with active non-infectious anterior, intermediate, and posterior uveitis, and compared these to 15 age and sex matched healthy controls. Results from manual gating were validated by automatic unsupervised gating using FlowSOM.

**Results:** Patients with uveitis displayed lower relative frequencies of Natural Killer cells and higher relative frequencies of memory T cells, in particular the CCR6+ lineages. These results were confirmed by automatic gating by unsupervised clustering using FlowSOM. We observed considerable heterogeneity in memory T cell subsets and abundance of CXCR3-CCR6+ (Th17) cells between the uveitis subtypes. Importantly, regardless of the uveitis subtype, patients that eventually required IMT in the course of the study follow-up exhibited increased CCR6+ T cell abundance before commencing therapy.

**Conclusion:** High-dimensional immunoprofiling in NIU patients shows that clinically distinct forms of human NIU exhibit shared as well as unique immune cell perturbations in the peripheral blood and link CCR6+ T cell abundance to systemic immunomodulatory treatment.

## Introduction

Non-infectious uveitis (NIU) is an umbrella term for a family of intraocular inflammatory conditions that collectively affect more than one in 1,000 individuals and account for ~10% of preventable blindness in Western countries (1, 2). This typically recurrent or chronic disorder may lead to poor visual outcome and low quality of life if undertreated (3, 4). Although corticosteroids (either local or systemically) remain the therapy of first choice, long term use is hampered by many side effects (5). Non-corticosteroid systemic immunosuppressive therapy (IMT) is often necessary to control inflammation and should be introduced when NIU is not controlled with corticosteroids alone or corticosteroid-sparing agents are required because of long term use or intolerance to corticosteroids (6). Consequently, there may be a considerable treatment delay in the patients who require IMT to control their uveitis. Since the need for IMT is not accurately predictable there is a clear need for early parameters that can identify patients who require IMT.

NIU is considered to be an immune-mediated condition driven by innate and adaptive immune activation (7–9). Although various of leukocyte subsets have been associated with NIU, autoreactive T cells are the protagonists in murine models of uveitis (9). Several flow cytometry studies in blood or eye fluid (i.e., aqueous humor or vitreous fluid) of NIU patients have revealed increased numbers of inflammatory T cell populations that typically produce Interleukin (IL)-17 (e.g., T helper (Th) 17 cells) and Interferon (IFN)-γ (e.g., Th1 cells) (10–12). Both Th17 and Th1 cells are considered to drive uveitis in animal models, yet in humans the evidence of their direct contribution to eye pathology remains circumstantial (13, 14). In addition, changes in the composition of principal subsets of human myeloid and plasmacytoid dendritic cells were observed in the serum of NIU patients and linked to disease activity (15, 16). What is more, IMT has been shown to selectively affect the phenotype or the function of specific leukocyte populations in blood (e.g., Th17 cells) which suggests that the frequency of these subsets in blood may be different in patients who require IMT (17–19). To our knowledge, studies that phenotype the majority of circulating immune cell subsets in three distinct clinical forms of (active) NIU are scarce and no studies have explored the potential link between the overall composition of blood cells and the need for IMT in this disease.

Here, we conducted a high-dimensional profiling of myeloid and lymphoid cell populations of patients with three archetypical types of human non-infectious uveitis. Using clinical data acquired during follow-up of the patients, we show that the high-dimensional immune profiling can identify patients who require IMT.

## Materials and methods

### Patients and patient material

This study was conducted in compliance with the Helsinki principles. Ethical approval was requested and obtained from the Medical Ethical Research Committee in Utrecht and all patients signed written informed consent before participation (Study number #NL46874.041.14).

We collected heparinized venous blood from a total of 30 adult patients with HLA-B27-associated Acute Anterior Uveitis (AU, *n* = 10), Idiopathic Intermediate Uveitis (IU, *n* = 9) or Birdshot Uveitis (BU, *n* = 11). Patients were seen at the outbound patient clinic of the uveitis center of excellence at the department of Ophthalmology of the University Medical Center Utrecht between July 2014 and July 2015. All patients had active uveitis [new onset (*n* = 11) or relapse (*n* = 19)] at the time of sampling. Activity was assessed by an experienced ophthalmologist. Uveitis was deemed active if there were clinical complaints in combination with one of the following features (new onset or an increase according to guidelines): anterior chamber cells (AU), vitritis (IU), cystoid macular edema (CME) on optical coherence tomography (OCT) or fluorescence angiography, or vasculitis or papillitis on fluorescence angiography (BU/IU) (20, 21). None of the patients had a related systemic auto-inflammatory or autoimmune disease, nor did they receive systemic immunomodulatory treatment in the last 3 months with the exception of a low dose of oral prednisolone (≤10 mg) for 1 BU patient. Of the 19 patients with recurrent disease eight had previously used systemic corticosteroids and four of these had also been treated with other immunosuppressants (including the BU patient receiving low dose prednisolone mentioned before).

Uveitis was classified and graded in accordance with the *Standardization of Uveitis Nomenclature* (SUN) classification (20). Each patient underwent a full ophthalmological examination by an uveitis specialist and routine laboratory screening, including erythrocyte sedimentation rate, renal and liver function tests, serum angiotensin converting enzyme (ACE), and screening for infectious agents (e.g., syphilis, Borrelia, TB) in blood. A chest X-Ray was performed to exclude Sarcoidosis. All patients with BU were HLA-A29 positive in the presence of characteristic birdshot lesions and all patients with AU were HLA-B27 positive. Fifteen age and sex matched anonymous blood donors with no history of ocular inflammatory disease served as healthy controls (HC). Medical records of uveitis patients were reviewed for demographic information. Follow up data were collected on the development of uveitis related complications [e.g., CME, the development of ocular hypertension (defined as intraocular pressure >21 mm Hg without optic nerve damage or visual field abnormalities but requiring therapeutic intervention)] and the use of systemic immunomodulatory therapy (IMT) (*n* = 23, with complete data). For two (BU) patients follow-up data were unavailable. IMT was defined as the use of any systemic immunosuppressive agent (i.e., DMARD, biological etc.) other than oral or intravenous corticosteroid therapy. The necessity of IMT was mostly based on persistent uveitis despite local corticosteroid therapy. In three cases, IMT was necessary to replace periocular steroids because it resulted in high intraocular pressure. The details of the study cohort are shown in Table 1.

**Table 1:** Characteristics of the cohort investigated in this study.

		**AU**	**IU**	**BU**	**HC**	***p*-value**
*N*		10	9	11	15	NA
Male		30%	22%	36%	40%	0.86^*^
Age in years; mean ± SD		44.8 ± 18.4	39.3 ± 14.1	53.4 ± 12.7	41.9 ± 10.0	0.11^**^
Disease duration in years; median (range)		5.7 (0.1–39.3)	3.7 (0.2–20.0)	4.5 (0.2–15.1)	NA	0.19^***^
New onset; *n* (%)		1 (10%)	4 (44%)	8 (73%)	NA
Follow-up after sampling in years; median (range)		2.1 (0.2–3.2)	2.8 (1.4–3.4)	2.7 (0.0–3.4)	NA	0.43^***^
Need for IMT^A^; *n* (%)		5 (50%)^B^	2 (22%)	8 (73%)^D,E^	NA
First	Methotrexate	5 (50%)	0	8 (73%)	NA
	Azathioprine	0	2 (22%)^C^	0	NA
Switch or addition	Mycophenolate mofetyl	0	0	2 (18%)	NA
	Mycophenolic acid	0	0	2 (18%)	NA
	Adalimumab	0	0	3 (27%)	NA

*BU, Birdshot uveitis; AU, HLA-B27 associated anterior uveitis; HC, healthy control; IU, idiopathic intermediate uveitis; NA, not applicable*;

* *Fishers exact test*;

** *Wilcoxon rank-sum test*;

*** *Kruskal–Wallis test*.

A *The use of any non-corticosteroid systemic immunosuppressive agent*.

B *Reasons for starting IMT: therapy resistance (n = 3), persistent activity and steroid response (i.e. secondary rise of intraocular pressure) to parabulbar injections (n = 2)*.

C *Reasons for starting IMT: persistent CME and steroid response to parabulbar injections (n = 1), persistent vasculitis (n = 1)*.

D *Reasons for starting IMT: Papillitis or vasculitis with or without CME (n = 6), CME resistant to parabulbar steroids (n = 1), persistent low-grade activity with visual field defects (n = 1)*.

E *From 2/11 BU patients follow-up is missing and treatment status is unknown*.

### Flow cytometric phenotyping

Seven to 10 million peripheral blood mononuclear cells (PBMCs) were isolated from heparinized venous blood by standard ficoll gradient centrifugation immediately after blood withdrawal and stored in liquid nitrogen. Patient and control PBMC samples were isolated and frozen for storage in a total of 29 batches. For flow cytometry analysis the nitrogen stored PBMCs were quickly thawed and washed once in ice cold FACS Buffer (1% bovine serum albumin (BSA) and 0.1% sodium azide in phosphate buffered saline). To account for potential between-run variability, nitrogen stored PBMC samples were randomized in 10 experiments including 4–5 samples per experiment. Per batch, thawed PBMC cells for each sample were divided for staining with five multi-color antibody panels; a 12-marker T-helper-panel (250,000 cells for staining, 100,000 events for acquisition), a 12-marker dendritic cell-panel (2.5 million cells for staining, 700,000 events for acquisition), a T regulatory cell panel based on 7 markers, a 11 marker B-cell panel (both 400,000 cells for staining, 100,000 events for acquisition) and a 11-marker T cell intracellular cytokine panel (750,000–1,500,000 cells for staining, entire samples measured for acquisition). These panels contained 37 unique protein markers in total (see for details Table S1). If not enough viable cells were available after thawing the samples, we did not include the B cell and Treg panel (*n* = 15 and *n* = 10 samples). The respective gating strategy used for each panel is outlined in each respective figure and Figures S1, S2.

For the T cell (intracellular) cytokine panel, PBMCs were first incubated for 4 h with RPMI-1640 (10% Fetal calf serum) and *Phorbol 12-myristate 13-acetate* (PMA), *Ionomycin* calcium salt and BD GolgiPlug (BD Biosciences, San Jose, CA, USA).

For the other panels, cells were incubated at room temperature (15 min) with 5% mouse serum to minimize non-specific binding of antibodies. Cells were then washed and suspended in FACS buffer after which they were incubated (20 min., 4°C) in the dark in V-bottomed plates with Brilliant Stain Buffer (BD, #563794) and the fluorescently-conjugated antibodies (Table S1). For intracellular staining (Treg-panel and intracellular cytokine panel), cells were subsequently washed, fixed and permeabilized with a fixation and permeabilization solution according to the manufacturer's instructions (eBioscience, San Diego, CA, USA), and stained (30 min, 4°C) with the appropriate antibodies (Table S1) in the dark.

**Figure 1:**
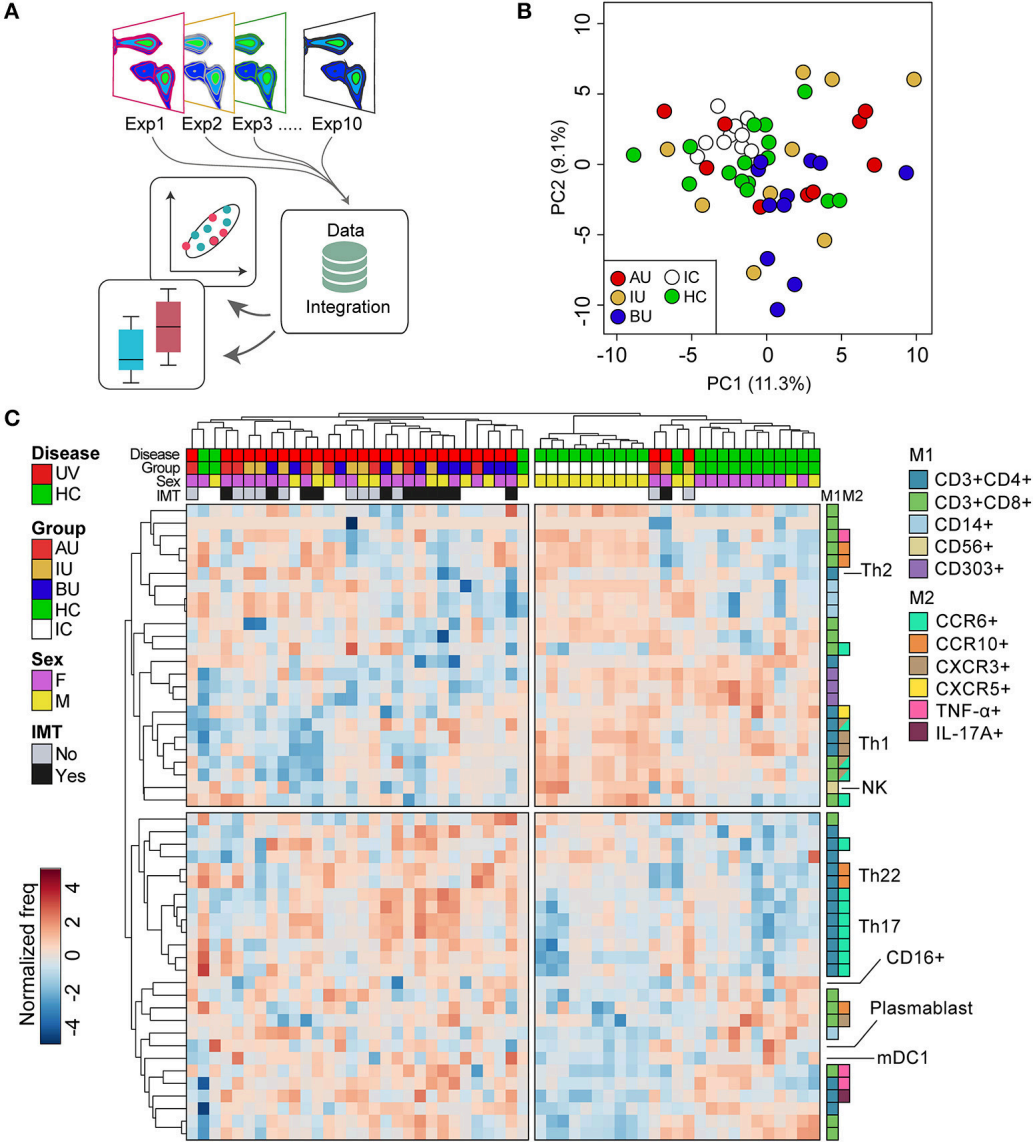
The overall blood leukocyte composition is changed in non-infectious uveitis **(A)**. We performed high-dimensional immune profiling of blood of non-infectious uveitis and control samples by multi-color flow cytometry in 10 independent experiments that were integrated for meta-analyses. **(B)** Principal component analysis (projection onto first two principal components) of the combined flow cytometry data of the 45 samples of this study, including the “internal control” (i.e., an aliquot from a single healthy donor isolated and frozen at the same day) that was taken along for each experiment. The close clustering of the internal control samples across all experiments as shown in the projection of the first two components indicates low inter-experiment variability. **(C)** Heatmap of unsupervised hierarchical clustering of the 50 manually gated cell subsets which largest variability between the groups (based on the ANOVA). Heatmap colors represent the changes in proportion from relatively lower proportion (in blue) to higher proportion (in red). Dendrograms indicating the clustering relationships between disease groups and leukocyte populations are shown to the left and above the heatmap. The sample meta-data are indicated above and the leukocyte subsets contributing to these clusters are indicated on the right. M1: first color marker to define leukocyte population. M2: additional color marker to define chemokine receptor and/or cytokine production. Populations with the same M1 and M2 colors differ in additional (not highlighted) protein markers (e.g., CD161) or represent the same population in a different gated population (% of CD4+CD45RO+ vs. % of CD3). AU, HLA-B27 associated anterior uveitis; BU, Birdshot Uveitis; F, female; HC, healthy control; IC, internal control; IMT, requirement for systemic (non-corticosteroid) immunosuppressive therapy during follow-up; IU, idiopathic intermediate uveitis; M, male; PC, principal component; Th, T helper cell; TNF, tumor necrosis factor; UV, all uveitis patients combined.

Flow cytometric analyses were performed on the BD LSR Fortessa™ Cell analyzer (BD Bioscience, San Jose, CA, USA). Data were analyzed using FlowJo software (TreeStar inc. San Carlos, CA, USA). The inter-assay variation was determined by measuring the same normal control (called “internal control”) in each of the 10 different days (separate batches see Figure 1A). Across all 10 experiments, this sample revealed low inter-assay variation (relative standard deviation <15% for all leukocyte populations with >5% within any of the gates) between aliquots of the same sample. We performed principal component analysis and hierarchical clustering on normalized frequency data of >100 leukocyte populations for NIU patients and controls. Based on the internal control, principal component analysis revealed high consistency between the 10 independent experiments performed (Figure 1B). Live single cells were identified using manual gating (Figure S1). A subpopulation was derived from its parent and grandparent population as indicated in Table S2. The following markers were excluded for further analysis based on suboptimal performance (insufficient discriminative capacity by fluorescent intensity across the experiments compared to fluorescent intensity data obtained for the antibodies during optimization of the panels; CCR4 in the T helper panel, and IgD, IgG, IgA in the B cell panel. In 2/10 experiments (NIU patients *n* = 6, HC *n* = 4) CCR6 was excluded for further analysis in these patients due to technical errors (consequently, a total of 23 patients—with appropriate follow-up were used for IMT analysis). “Fluorescence-minus-one” (FMO) control experiments were performed for the intracellular cytokine and surface staining before the start of analysis to determine the cut-off between positive and negative cell populations.

### Statistical approaches

Statistical analyses on manually gated data were performed in SPSS version 21.0 (SPSS Inc., Chicago IL, USA) or MetaboAnalyst v4.0 (22). To categorize patients and controls into groups with similar leukocyte composition in blood, data from all 10 independent experiments were integrated, and subjected to principal component analysis and hierarchical clustering. For cluster analyses the data were log10-transformed, auto-scaled and compared using a correlation-based (Pearson's) dissimilarity measure, which considers two objects to be similar if their features are highly correlated regardless of their magnitudes and the average linkage method (combine clusters with the smallest average linkage distance). For hierarchical clustering, missing values for each leukocyte population were imputed with the median proportion (%) of all samples. The Wilcoxon rank-sum test or Kruskal–Wallis test with the Dunn's *post-hoc* test were used to assess group differences.

### FlowSOM

To technically validate our findings, we performed unsupervised clustering of single cell gated data from the T helper panel by Bioconductor package *FlowSOM*. FlowSOM uses self-organizing map (SOM) algorithm to cluster cells into distinct clusters. Before executing the SOM algorithm, the flow cytometry data were subjected to logicle transformation and expression of each marker is scaled according to Z-score normalization. We first pooled all the cells together from each sample and performed 2,000 iterations to train the SOM (grid size = 100). We then clustered all cells into 100 distinct clusters based on their similarities in high-dimensional space (i.e., considering the relative fluorescent intensities of 11 protein markers of the T helper panel simultaneously (minus CCR4, see methods) (23). Cluster annotation was performed using consensus hierarchical clustering, as implemented in the ConsensusClusterPlus R package (24). The number of meta-clusters was determined by FlowSOM algorithm using the elbow criterion allowing for a maximum of 90 clusters. To prevent overfitting of putative rare cell populations, we did not consider clusters with a mean proportion of <0.1% for further analyses.

Meta-analysis of frequency data across all 10 experiments for the 30 uveitis patients and the 15 unaffected controls were reported for subsets with an altered median frequency in the combined dataset below a type I error of 5% (*p* < 0.05) and considered significant when manually gated cell populations and clusters identified (FDR <1%) by FlowSOM exhibited similar phenotypes (Table S2).

## Results

We deeply phenotyped the circulating immune cells of patients with three distinct types of active non-infectious uveitis (NIU) and healthy controls by integrating the results of 10 (randomized) flow cytometry experiments, which comprise five unique marker panels (Figure 1A). We first conducted principal component analysis (PCA) of the combined manual gated data to investigate the inter-experiment variation. PCA revealed close clustering of the internal control samples measured in each of the 10 independent experiments (Figure 1B). The close clustering of the internal control samples indicates consistent results across all 10 experiments between aliquots of the same sample and justifies combining all data for further analysis. The details of the subsets identified by manual gating are given in Table S2. Unsupervised hierarchical clustering of the 50 leukocyte subsets with the largest variance between the groups revealed two overarching sample clusters: 1 cluster of 30 samples of which 90% were uveitis samples, and a second cluster that contained mostly controls samples (88% of samples). This analysis revealed that overall it was possible to distinguish uveitis patients from healthy controls considering the abundance of multiple leukocyte populations in blood, (Figure 1C), predominantly T cells (for detailed information see Table S2).

### Shared and uveitis subtype specific changes in the T cell repertoire in patients with non-infectious uveitis

Most perturbations were observed in the T cell panel. T cells (CD3^+^) were divided into CD4 expressing T helper cells (Th) and CD8 expressing cytotoxic T cells (Tc). Using the expression of the surface markers CD45RO and CD27 on CD4^+^ and CD8^+^ T cells, we categorized naïve (T_N_, CD27^+^CD45RO^−^), effector (T_E_, CD27^−^CD45RO^−^), and memory (T_M_, CD27^+/−^CD45RO^+^) phenotypes (Figure S3).

### CD4+ T (helper) cells

**Figure 2:**
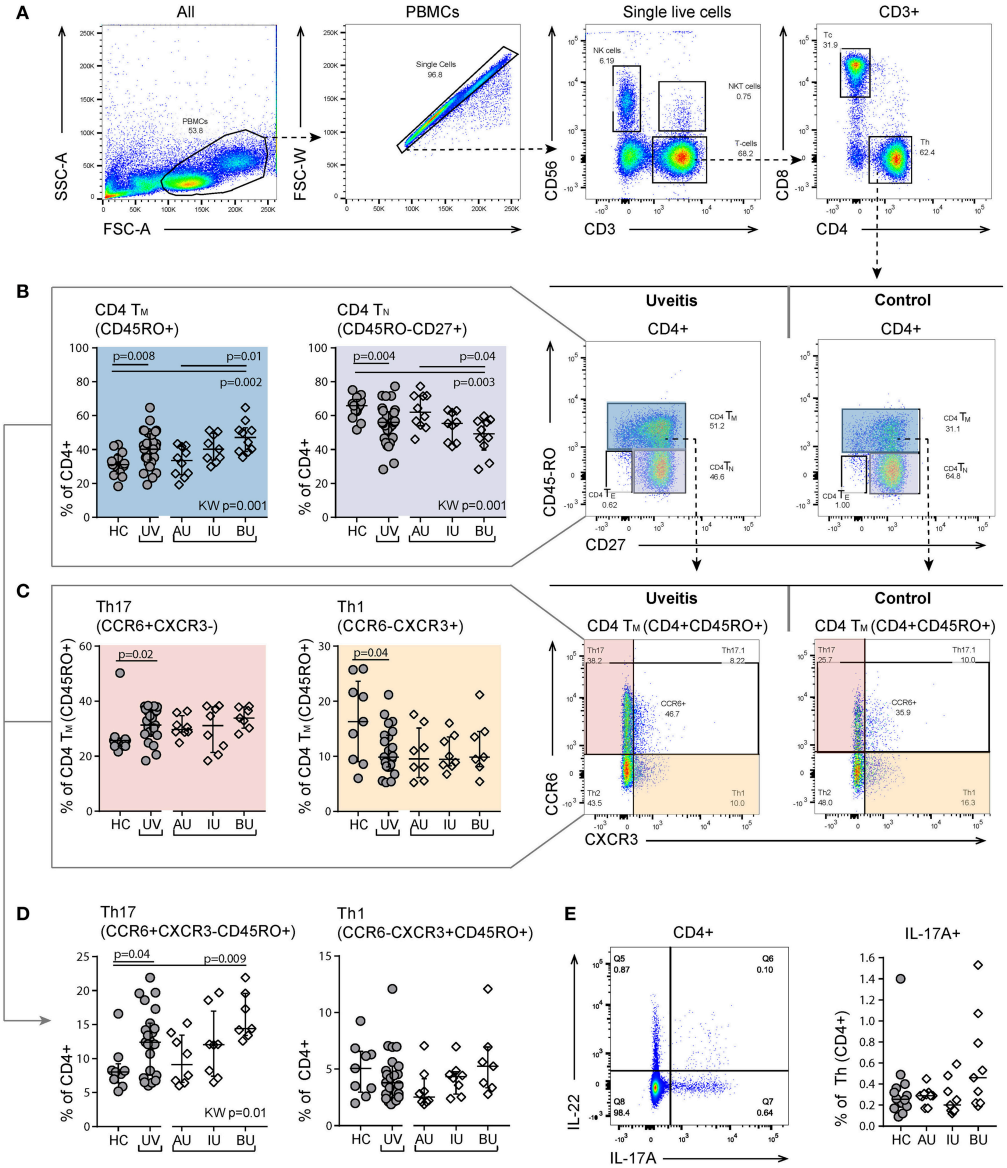
Heterogeneity in memory T cell subsets and abundance of CXCR3-CCR6+ (Th17) cells between uveitis subtypes. **(A)** Gating strategy of a representative sample used to identify T helper (Th. CD3^+^CD4^+^) and cytotoxic T cells (Tc. CD3^+^CD8^+^). **(B)** Shift in proportion from naïve to memory T helper cells in uveitis patients compared to controls. The proportion of CD4^+^T_N_ and CD4^+^T_M_ cells shows heterogeneity between the uveitis subtypes and the observed difference between uveitis and healthy controls is mainly driven by BU patients. **(C)** Within the CD4^+^T_M_ there is an increase of Th17 (CCR6+CXCR3-) and a decrease in Th1 (CCR6-CXCR3+) cells in uveitis patients compared to healthy controls. **(D)** BU displayed an increase in Th17 cells within the total CD4^+^ T cell population compared to the other patient groups. **(E)** left: gating strategy used to identify IL-17A expressing CD4+ T helper cells using the intracellular cytokine panel. Right: percentage of IL-17A+ cells within the total CD4^+^ T cell population. The gating of intracellular cytokines (including IL17A) from CD4 T cells in given in more detail in Figure S2. Bars indicate the median and interquartile range. *P*-values between HC and UV are from Wilcoxon rank-sum test, *p*-values between uveitis subgroups are from Kruskal–Wallis (KW) test with *post-hoc* Dunn's correction for multiple testing. AU, HLA-B27 associated anterior uveitis; BU, Birdshot uveitis; HC, healthy control; IL, interleukin; IU, idiopathic intermediate uveitis; T_E_, effector T cell (CD45RO-CD27-); Th, T helper cell (CD3+CD4+); T_M_, memory T cell (CD45RO+); T_N_, naïve T cell (CD45RO-CD27+); PBMC, peripheral blood mononuclear cell; TNF, tumor necrosis factor; UV, all uveitis patients combined.

In CD4^+^ T (helper) cells, we noted a marked change in the proportion of naïve and memory cells in BU patients, but not for AU patients (Figures 2A,B). The frequency of CD4^+^ T_M_ cells is known to be associated with age and we found a similar correlation between CD4^+^ T_M_ cells and age (rho = 0.4, *p* = 0.009) (25). To decouple the effect of age and disease, we used a linear regression model and found that the increased proportion of CD4^+^ T_M_ cells was independently associated with uveitis (*p* = 0.02). Within the CD4^+^ T_M_ subset, uveitis patients showed a significant increase in the proportion of CCR6^+^CXCR3^−^ cells (Th17) and a decreased proportion of CCR6^−^CXCR3^+^ (Th1) cells (Figure 2C, *p* = 0.02 and *p* = 0.04 respectively). BU displayed an increase in CCR6^+^CXCR3^−^ cells (Th17) within the total CD4^+^ T cell population compared to the other patient groups (Figure 2D). A similar trend was noted when considering IL-17-producing CD4^+^ T cells (Figure 2E). We found no specific enrichment for CD161^+^ Th17 lineage cells in uveitis patients (Table S2) (26, 27).

**Figure 3:**
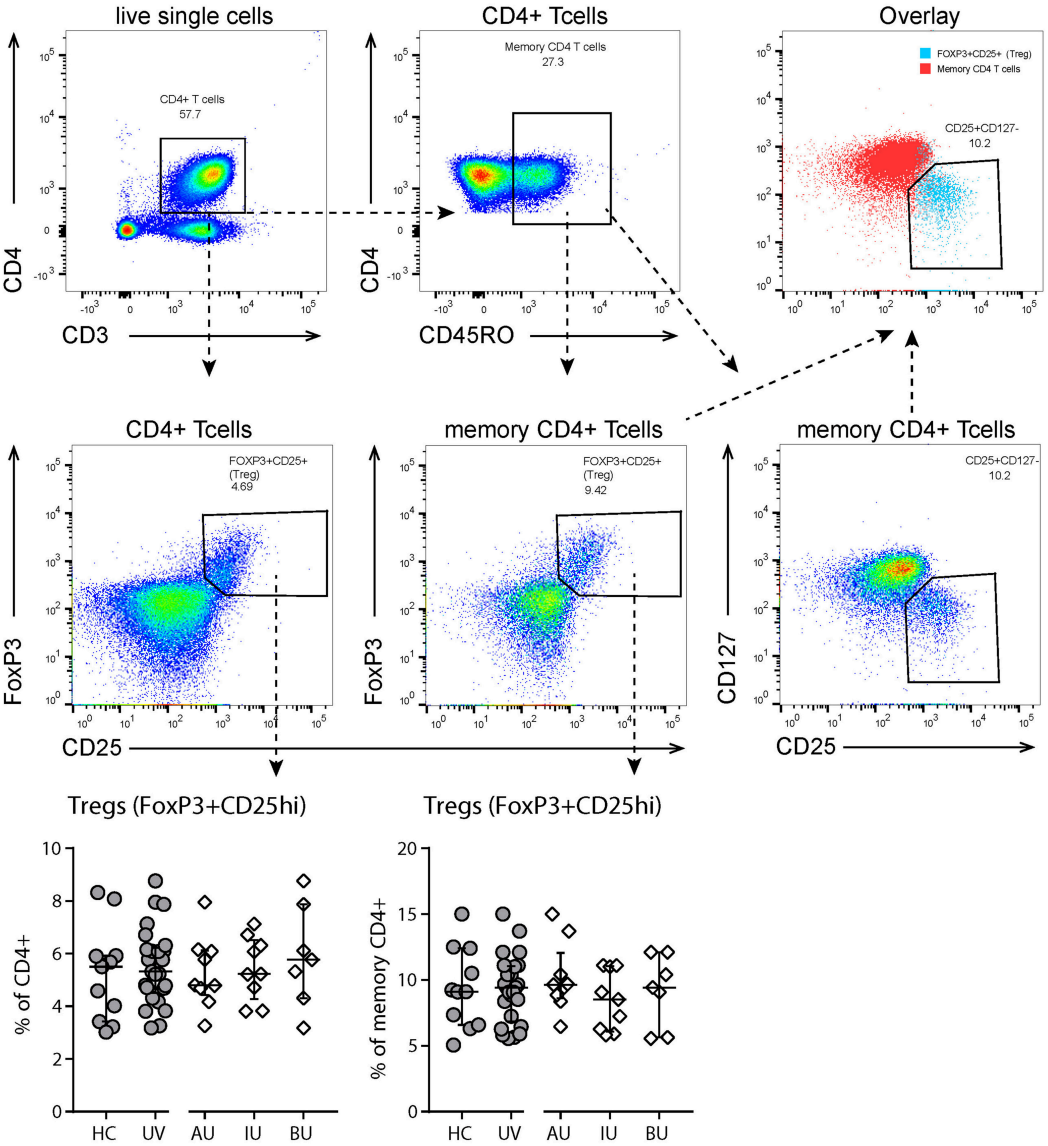
Flow cytometry analysis of T regulatory cells. **Top and second row:** gating strategy used to identify T regulatory (Treg, CD25highFoxP3+) cells. Live single cells are gated as indicated in Figure S1. **Bottom row:** levels of circulating Tregs as % of (memory) CD4+ T cells. All Tregs are also CD127- (see “overlay” plot). Bars in scatter plots indicate median, error bars indicate interquartile range. Abbreviations: AU, HLA-B27 associated anterior uveitis; BU, Birdshot uveitis; HC, healthy control; IU, idiopathic intermediate uveitis; UV, Combined uveitis samples.

Finally, we observed a significant decrease in CD4^+^ T_m_ CD27^+^CXCR5^+^ cells (i.e., follicular T helper cells) in uveitis patients (Table S2). There were no changes in the percentage of regulatory (CD3^+^CD4^+^CD25^hi^ CD127^low^FoxP3^+^) T cells (Figure 3).

### CD8+ T cells

**Figure 4:**
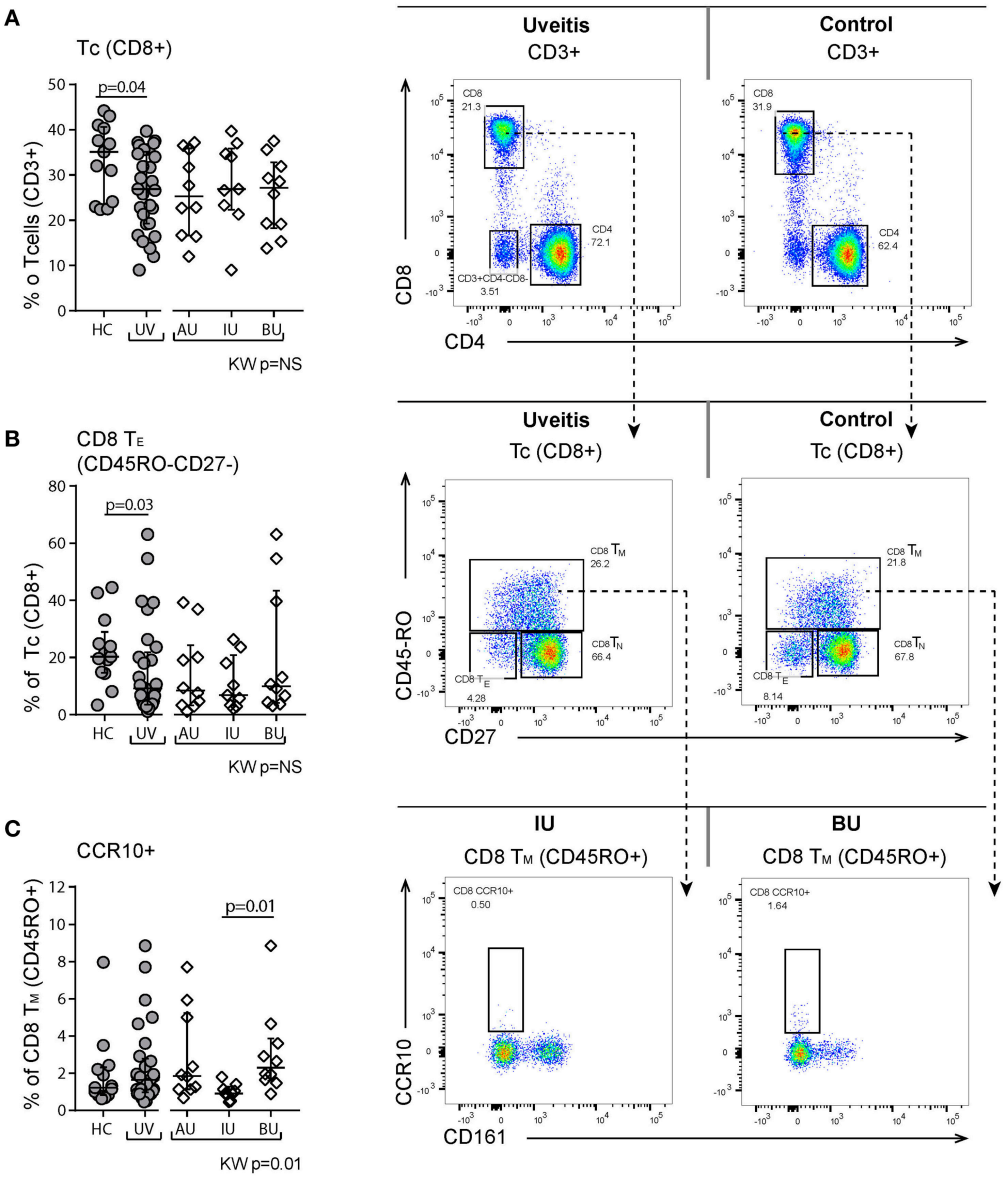
Flow cytometry of peripheral blood mononuclear cells shows changes in the proportion of CD8+ T cell subsets. **(A)** Decrease of CD8+ T cells in uveitis compared to healthy controls. Representative patient and control sample. CD3+ cells are gated from live single cells, as is indicated in Figure S1. **(B)** Decrease in the CD45RO-CD27- T_E_ population. **(C)** CCR10+ CD8+ T_M_ cells are significantly higher in BU compared to IU. Bars in plots indicate median, error bars indicate interquartile range. *P*-values between HC and UV are from Wilcoxon rank-sum test, *p*-values between uveitis subgroups are from Kruskal–Wallis (KW) test with *post-hoc* Dunn's correction for multiple testing. AU, HLA-B27 associated anterior uveitis; BU, Birdshot uveitis; HC, healthy control; IU, idiopathic intermediate uveitis; Tc, cytotoxic Tcell; Th, Thelper cell; T_E_, effector Tcells (CD45RO-CD27-); T_N_, naïve (CD45RO-CD27+); T_M_, memory (CD45RO+); UV, Combined Uveitis samples.

Within the (CD3^+^) T cell populations, the ratio of CD4^+^ to CD8^+^ T cells was higher in uveitis patients (ratio [range] = 2.5 [1.4–9.4] vs. 1.5 [1.1–3.3], *p* = 0.03), due to a reduction in CD8^+^ cells in the CD3^+^ population (Figure 4A). This decrease in the proportion of CD8^+^ T cells was primarily caused by a decrease of CD8^+^ T_E_ cells in NIU patients (Figure 4B). Apart from the differences between NIU and healthy controls, we found that CCR10^+^ CD8^+^ T_M_ cells were significantly increased in BU patients compared to IU patients (median % [range] IU = 0.9 [0.7–1.3], BU = 2.3 [1.6–3.9], *p* = 0.01, Figure 4C).

### Dendritic cells, NK cells and B cells

**Figure 5:**
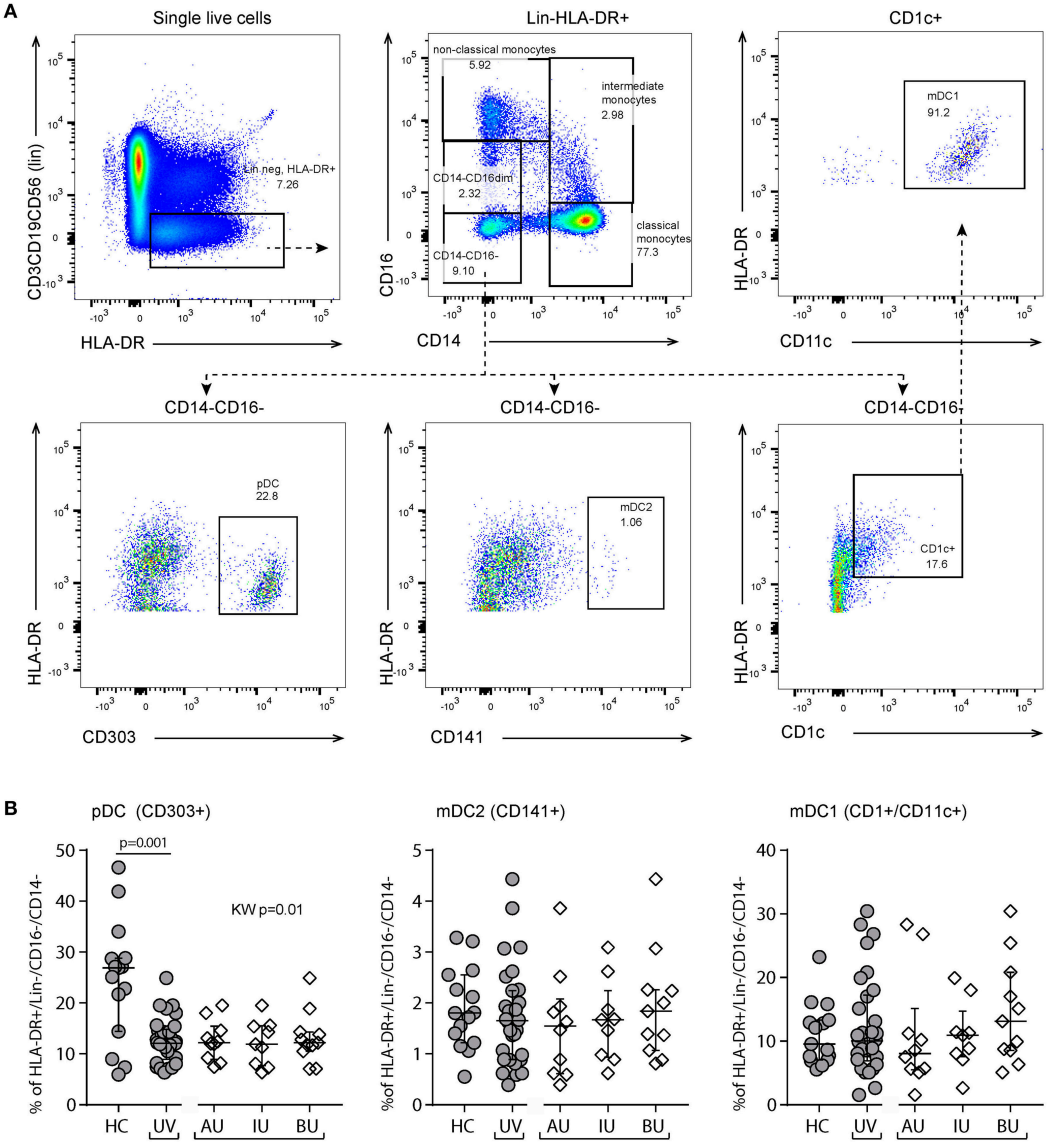
Flow cytometry of peripheral blood mononuclear cells shows a decrease of plasmacytoid dendritic cells in non-infectious uveitis. **(A)** Gating strategy used to identify dendritic cell subsets (representative sample). Live single cells are gated as indicated in Figure S1. **(B)** The frequency of circulating plasmacytoid and myeloid dendritic cell subsets as percentage of the HLA-DR^+^Lineage (CD3,CD19,CD56)^−^CD16^−^CD14^−^ cells. Bars in plots indicate median, error bars indicate interquartile range. *P*-values between HC and UV are from Wilcoxon rank-sum test, *p*-values between uveitis subgroups are from Kruskal–Wallis (KW) test with *post-hoc* Dunn's correction for multiple testing. AU, HLA-B27 associated anterior uveitis; BU, Birdshot uveitis; HC, healthy control; IU, idiopathic intermediate uveitis; KW, Kruskal–Wallis test; mDC, myeloid dendritic cell; pDC, plasmacytoid dendritic cell; UV, Combined Uveitis samples.

**Figure 6:**
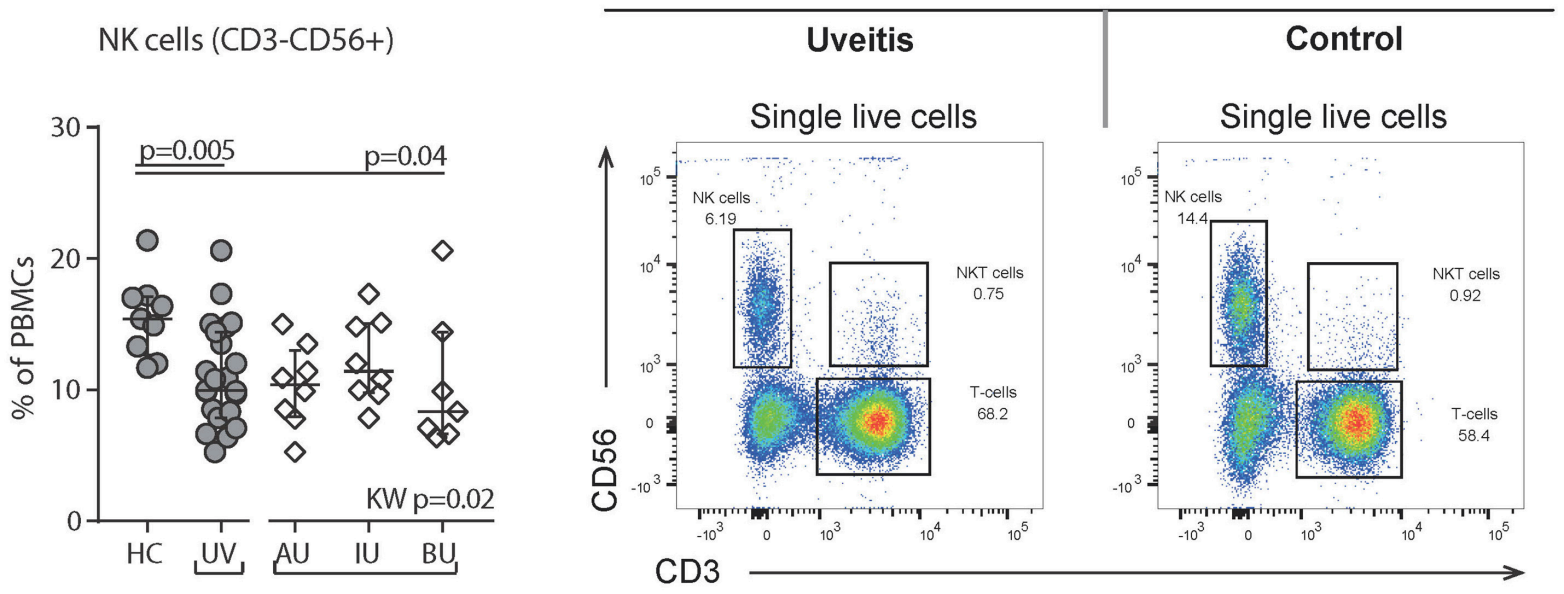
Flow cytometry of peripheral blood mononuclear cells shows a decrease of Natural Killer (NK) cells in uveitis. **Left:** levels of circulating NK (CD56+CD3-) cells as % of PBMCs. **Right:** gating strategy used to identify NK cells. Live single cells are gated as indicated in Figure S1. Bars in plots indicate median, error bars indicate interquartile range. *P*-values between HC and UV are from Wilcoxon rank-sum test, *p*-values between uveitis subgroups are from Kruskal–Wallis (KW) test with *post-hoc* Dunn's correction for multiple testing. AU, HLA-B27 associated anterior uveitis; BU, Birdshot uveitis; HC, healthy control; IU, idiopathic intermediate uveitis; PBMC's, peripheral blood mononuclear cells; UV, Combined Uveitis samples.

We observed a decrease in the proportion of plasmacytoid dendritic cells (pDCs, CD303^+^CD14^−^CD16^−^HLA-DR^+^Lin^−^, *p* = 0.001, Figure 5) and natural killer cells (NK, CD56^+^CD3^−^, *p* = 0.005, Figure 6), and an increased proportion of CD19^+^ B cells (Table S2). The proportion of monocyte or myeloid dendritic cell subsets was comparable between NIU and healthy controls (Table S2).

In summary, our observations from manual gating show that the composition of multiple immune cell subsets are altered in different types of NIU patients and that most changes are observed in the T cell repertoire.

### Automatic gating by FlowSOM confirms changes in T and NK cell phenotypes linked to non-infectious uveitis

**Figure 7:**
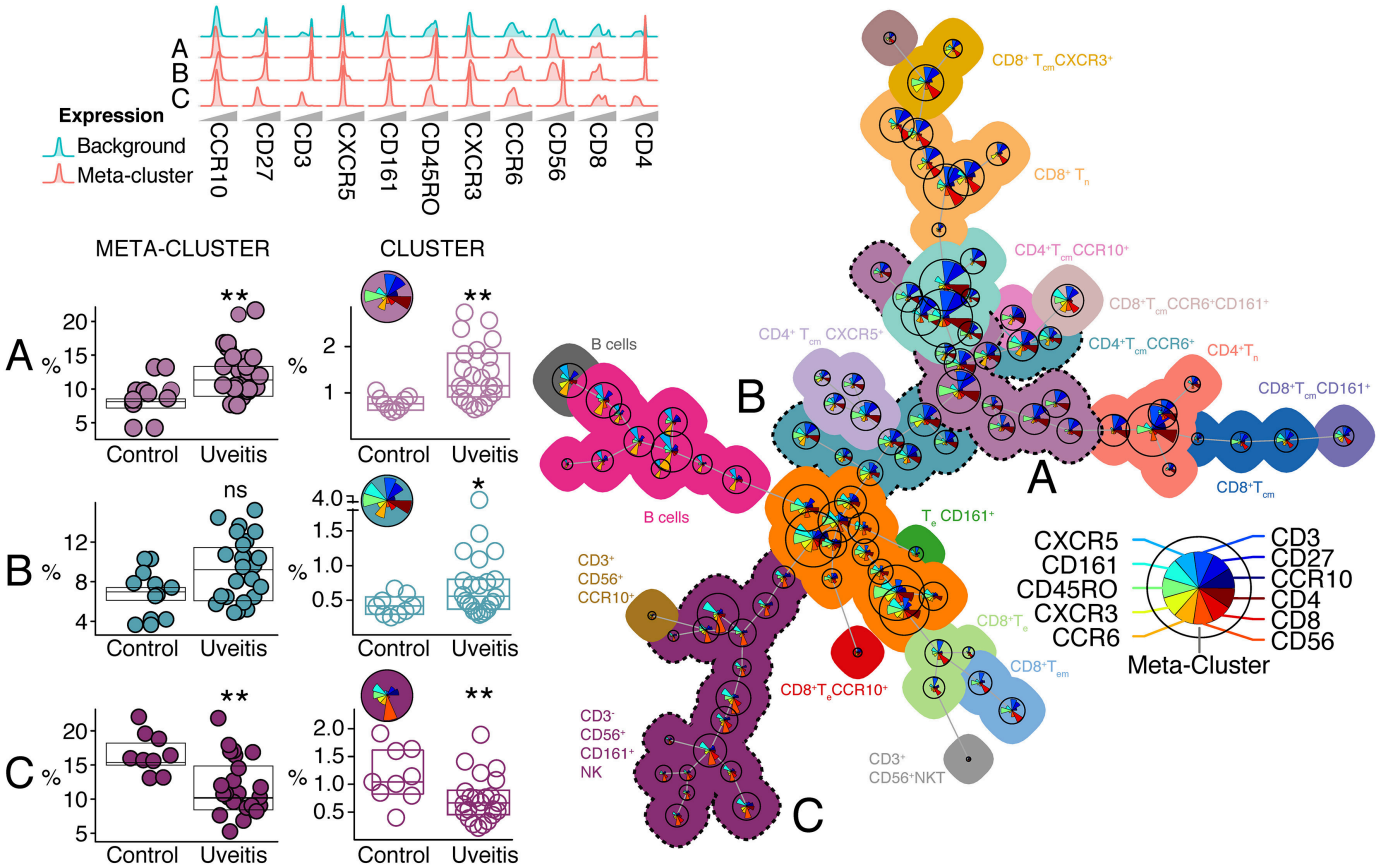
Automatic gating by unsupervised clustering using FlowSOM identifies cell (meta) clusters associated with non-infectious uveitis. Using FlowSOM, we clustered all individual cells within the single-cell gates of all samples into 100 distinct clusters based on the surface protein expression. Using unsupervised clustering, the 100 clusters were clustered into 22 meta-clusters of different cell types and organized in the minimal spanning tree on the right. The meta-clusters are represented by unique colors (all meta-cluster comparisons between uveitis and controls are provided in Figure S3). For each cluster (i.e., cell population), pie charts indicate the relative expression for each of the different surface markers and the pie size corresponds to the average size of the population in the samples. Three clusters and their associated meta-clusters (A–C) are indicated. *P*-values between HC and UV are from Wilcoxon rank-sum test (*P < 0.05, **P < 0.01, ns, non-significant). For these three meta-clusters, the relative densities of normalized expression of the surface protein markers for from the T cell panel are indicated above and for each associated cluster the pie charts provide the relative expression of each surface marker.

To technically validate our findings, we further explored the T helper cell panel by automatic gating using *FlowSOM* (23) that performs multivariate clustering of cells based on the self-organized map (SOM) algorithm and categorizes cells into relevant meta-clusters based on their surface markers. Using FlowSOM, we clustered all individual cells into 100 distinct clusters based on the surface expression marker proteins. Using unsupervised approaches, we further clustered these 100 clusters into 22 meta-clusters that are outlined in the minimal spanning tree shown in Figure 7 (for details see Figure S4 and methods). High-dimensional automated gating identified three common (>0.1% abundance) meta-clusters that were linked to uveitis and corroborate the subsets determined by manual gating. We observed a significant increase in the proportion of *meta-cluster A*, that contained memory CD4^+^ T cells expressing CD45RO and CD27 (28) in uveitis patients (Figure 7). Analysis by FlowSOM further confirmed a significant decrease in a cluster reminiscent of NK cells (CD3^−^CD56^+^, *meta-cluster C*, Figure 7). We also noted a CD4^+^ memory meta-cluster (*meta-cluster B*) that contained subsets of Th17 enriched (CXCR3^−^CCR6^+^) clusters that were significantly increased in NIU (Figure 7). In short, the leukocyte populations associated to NIU by FlowSOM are similar to the populations identified by manual gating.

### Higher abundance of CCR6+ T cells in patients that require systemic immunosuppressive treatment

**Figure 8:**
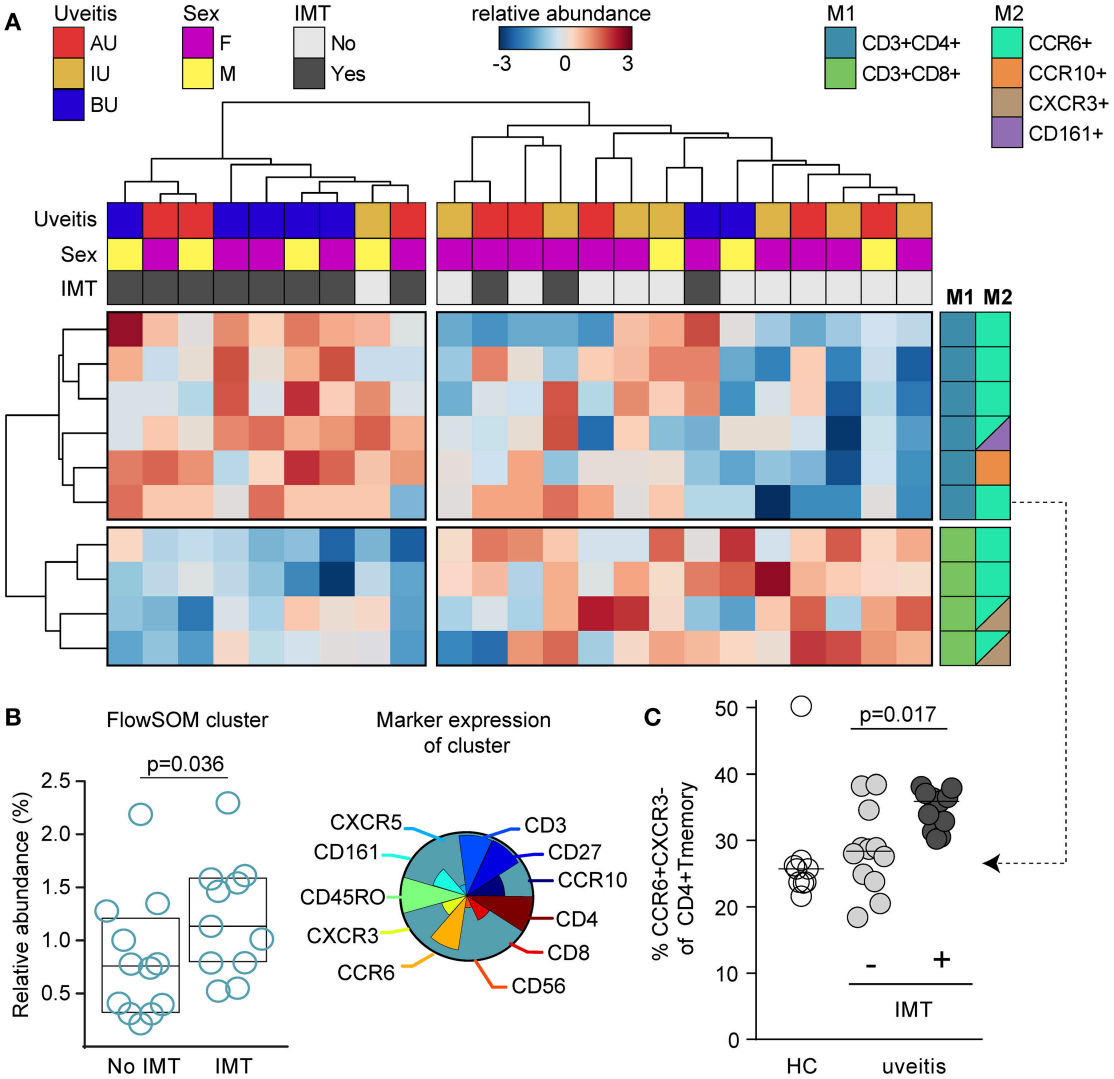
CCR6+ T cells are increased in patients that need systemic immunomodulatory treatment. **(A)** Unsupervised hierarchical clustering of the top 10 T cell subsets in 23 patients with complete data distinguishes two clusters of patients that are characterized by altered proportion of CD4^+^ and CD8^+^ T cell populations and differential requirement of IMT during follow-up. Heatmap colors represent the relative difference in proportion from relatively low (in blue) to high (in red) for each manually gated population. Data were normalized using quantum normalization and log transformation. Data are scaled using Auto scaling. The sample meta-data and the leukocyte subsets contributing to these clusters are indicated. M1, first color marker to define CD4 or CD8 expression in T cell populations; M2, additional color marker to define chemokine receptor or CD161 expression for each T cell population. **(B)** FlowSOM analysis identified a cluster from meta-cluster B with significantly higher abundance in patients that required IMT during follow-up before commencing therapy. This IMT associated FlowSOM cluster has a phenotype consistent with Th17 cells (CD3+CD4+CD45RO+CXCR3-CCR6+). The pie chart indicates the relative expression for each of the different surface markers that is expressed on this cluster. The scatterplot (left) depicts the proportion of this cluster within the live single cell gate that was fed into the FlowSOM algorithm. Bars indicate median with interquartile range. **(C)** Scatterplot of manually gated Th17 (CXCR3-CCR6+ of CD4+ T_M_ cells) cells shown for controls and the treatment groups. Bars indicate the median. *P*-values are from Wilcoxon rank-sum test. AU, HLA-B27 associated anterior uveitis; BU, Birdshot uveitis; F, female; HC, healthy control; IMT, requirement for systemic (non-corticosteroid) immunosuppressive therapy during follow-up; IU, idiopathic intermediate uveitis; M, male.

Building on the manual gating, we first explored if the composition of blood leukocytes was different between patients that subsequently required IMT during follow-up. In total, 15 patients (eight with new onset uveitis, seven with a recurrence) started IMT within the first year after sampling (median 0.2 months, range 0.0–12.1). We first subjected the manual gated data from the T helper cell panel to hierarchical clustering to investigate the T cell composition in 23 NIU patients with complete data (see section Materials and Methods). Unsupervised clustering distinguished two overarching groups; the first cluster contained near exclusively patients (8/9, 89%) that during follow-up required IMT and the second cluster contained mostly patients that did not require IMT during follow-up (14/17, 82%). The cluster associated with IMT was characterized by increased proportions of CD4^+^CXCR3^−^CCR6+ Th (17) populations and decreased proportions of CD8^+^CCR6+ T cell populations (Figure 8A). These findings indicate that patients that will require IMT are characterized by distinct composition of T cells in blood. To further support these observations, we used FlowSOM for head-to-head comparison of IMT+ and IMT- patients. Analysis by FlowSOM supported that a cluster of Th17 cells was significantly more abundant in patients who required IMT (Figure 8B, Figure S5). Using manual gating we could confirm that this Th17 population was more abundant in patients that require IMT during follow-up (Figure 8C). Two additional leukocyte clusters significantly differentiated between IMT+ and IMT- patients in the FlowSOM analysis (Figure S5), but these clusters showed low CD3 expression and represent populations other than T cells. In summary, patients with non-infectious uveitis that in the near future will be treated with IMT show increased Th17 cells in blood.

## Discussion

In this study, we demonstrated that patients with anatomically distinct types of non-infectious uveitis (NIU) exhibit shared and unique perturbations in circulating immune cell subsets, most notably, subtype-specific changes in the T cell repertoire. The overall changes in proportions of leukocyte subsets were modest, which is typically reported in flow cytometry studies of non-infectious uveitis (11, 16, 18). Importantly, patients that required systemic immunomodulatory treatment (IMT) after sampling had higher CD4^+^ populations expressing CCR6.

The pathogenic mechanisms underlying NIU remain incompletely understood, but are certainly immune-mediated (13, 29). In humans, multiple lines of evidence have linked type 17 immune responses (e.g., IL-17-producing T cells) to NIU (11, 12, 14, 30–33). In line with this, we observed an increase of (CCR6^+^CXCR3^−^) Th17 cells within CD4 T_M_ cells that was noticeable for all uveitis types. However, we observed a relatively large uveitis-subtype specific difference in memory CD4 (T_M_) cell proportion, which suggests that the involvement of T helper populations might be different between uveitis subtypes (34). The enrichment for memory CD4 T cells in BU contributed to a hitherto unappreciated larger Th17 fraction in CD4^+^ T cells in BU over AU. Although a large variety of clinical characteristics distinguish clinical phenotypes of NIU, the fast majority of molecular factors identified are shared across most types of NIU. Consequently, the current view on etiology considers the disease mechanisms of AU, IU, and BU largely identical. Here, we deliberately reported results per clinical diagnosis and identified shared, but also heterogeneity between the studied uveitis groups. We believe this heterogeneity in immune cell perturbations may reflect differences in pathophysiological mechanisms between uveitis subtypes that has hitherto been under appreciated. This is an important observation, because to date, studies reporting on increased Th17 cells in human NIU have not accounted for uveitis subtype specific differences, which might explain inter-study variability. Also, many studies lack of markers that differentiate between memory populations (e.g., define Th17 populations solely on CD4^+^IL-17^+^ cells).

There remains a lack of understanding which patients will require IMT for durable disease control (35). Here, we provide evidence that the proportion of CCR6^+^ T cells, which are enriched for Th17 cells, may harbor predictive capacity for the need of IMT. Interestingly, in our cohorts, there were eight patients that had a history of treatment with systemic corticosteroids (<3 months) before inclusion, four of whom had received IMT, but half of the patients with a history of systemic corticosteroids, and one out of four patients with a history of IMT did not need IMT after sampling. We would like to emphasize that the current study was designed for phenotyping the myeloid and lymphoid compartment and less suited for testing the clinical utility of the identified cell populations. For example, the personal wish of the patient for periocular steroids over systemic IMT is not taken into account. Therefore, prospective studies with larger cohorts and longitudinal sampling will be needed to substantiate these observations and determine their clinical impact. Since manual as well as unsupervised gating methods linked CCR6-expressing T helper cells to uveitis biology and treatment use, we suggest this T cell family should be a central subject of such studies. Interestingly, a recent study demonstrated that flow cytometry phenotyping of Th17 cells can potentially stratify patients to better match the choice of biological (e.g., *Ustekinumab*) after MTX resistance in patients with psoriatic arthritis, an inflammatory conditions associated with uveitis (36). In addition, immunophenotyping of Th17 populations may be particularly useful for identifying patients that may benefit from therapeutic drugs targeting the IL-23/IL-17 axis (e.g., *Ustekinumab*) or the Th17-related JAK-STAT-pathways (37–39). It is tempting to speculate on the outcome of previous anti-IL-17 trials in NIU if patients groups were matched according to their T cell repertoires (37).

High resolution phenotyping of T cell populations using a mass cytometry (e.g., CyTOF) to study a wide array of activation and signaling molecules might be of additive value to better define the T cell fingerprints of NIU, and improve prediction and choice of IMT for individual patients (40). Also, future studies should not be limited to T cell populations: we recently linked serum microRNA levels of NIU patients to decreased frequency of blood CD16^+^NK cells (41). Strikingly, we confirmed a decrease in the proportion of CD56^+^ NK cells in patients with active uveitis. NK cells [which can be roughly divided in CD16^+^CD56^dim^ and CD16^−^CD56^hi^ cells (42)] can directly infiltrate the eye during uveitis, perhaps by binding of their *Killer immunoglobulin-like* (KIR) receptors, a class of immune receptors that have been genetically linked to Uveitis (43, 44). Of note, Ustekinumab also targets IL-12, a cytokine elevated in uveitis serum and by which CD56^+^ NK cells are strongly activated (45).

We confirmed a decrease in the proportion of circulating plasmacytoid (p)DCs in human non-infectious uveitis, but were unable to ascertain a significant increase in CD1c-positive myeloid DCs (mDC1) (16, 46, 47). This is likely to be explained due to a different uveitis population. Other novel observations of interest include an increase in (CD19^+^) B cells and decrease in (CXCR5^+^CD27^+^) T follicular helper cells, a T helper subset linked to B cell function (48). Note, inhibition of B cells by rituximab has been shown to be effective for some types of NIU (49, 50). Most flow cytometry studies in NIU have focused on the frequency or function of regulatory T cells (Tregs) in blood (51, 52). In our study, we did not observe changes in the proportion of these cells which adds up to the conflicting results in the literature on this topic (11, 12, 18, 33, 53–55).

Though many studies report immune perturbations in the peripheral blood of uveitis patients it is important to realize that the immune changes that are found in the peripheral blood do not necessarily mirror the changes in the eye (32, 56, 57). The results of this study should be considered in light of the following limitations. We deliberately included only uveitis patients with active disease and free from systemic treatment at the time of sampling. Although these stringent selection criteria contributed to several unique observations, the overall small changes in leukocyte percentages in blood combined with the relatively small sample size hampered statistical power. However, by using unsupervised algorithms were able to validate our main observations in T cell and NK cell proportions.

The absence of CCR4 in our data may have hampered accurate Th2 frequency estimation, but Th17 and Th1 subsets are well-identified using CCR6 and CXCR3 (58–60). Although CCR6 can be expressed by other T cells (including B cells and dendritic cells), CCR6 expression on T cells is mediated by the Th17-lineage transcription factor RORγt, and CCR6+ T cells show over 100 fold increase in IL-17 production compared to CCR6-negative T cells (61–64). Therefore, CCR6 expression on T cells is often used to identify the Th17 subset (64–67).

Also, flow cytometry is limited in number of simultaneous markers (~12–16-colors per panel) and prevents deep phenotyping of cell status (e.g., activation, intra-cellular signaling) of cells. Although FlowSOM supported our gating strategy based on the here investigated markers, subsequent detailed phenotyping focusing on specific subsets (e.g., T and NK populations) or other subsets (e.g., innate-like lymphoid cells, neutrophils) may benefit from using mass cytometry (CyTOF) to obtain more in-depth information on the cell signatures that can predict disease outcome of NIU patients.

Finally, this study was conducted at a tertiary referral center. Therefore, our cohorts are more likely to include patients with complicated disease courses. This explains, for example, why half of our AU patients needed IMT, a uveitis subtype for which topical treatment is often sufficient (68).

In conclusion, NIU is characterized by changes in circulating immune cell subsets, particularly T cell populations that were associated with future IMT requirement. A deeper functional understanding of these observations will aid in optimizing timely therapeutic interventions of this potentially blinding disease.

## Author contributions

FV: acquisition of data, execution and interpretation of experiments and results, analysis of the data, drafting the manuscript. SH: designing the flow cytometry panels, acquisition of data, execution, and interpretation of experiments and results. RR: execution and interpretation of FlowSOM analysis, revising the manuscript. AP: interpretation of FlowSOM analysis, revising the manuscript. EL: acquisition of data, execution of experiments, revising the manuscript; MO: acquisition of data, execution of experiments, revising the manuscript. NtD-vL: acquisition and interpretation of data, revising the manuscript. SN: designing the flow cytometry panels, revising the manuscript. SI: acquisition and interpretation of data, revising the manuscript. JdB: study design, interpretation of experiments and result, supervisory support, revising the manuscript. TR: study design, interpretation of experiments and result, supervisory support, revising the manuscript. JK: study design, analysis of the data, interpretation of experiments and result, supervisory support, drafting, and revising the manuscript.

### Conflict of interest statement

The authors declare that the research was conducted in the absence of any commercial or financial relationships that could be construed as a potential conflict of interest.
