# Supplementary figures and images for: High-Dimensional Profiling Reveals Heterogeneity of the Th17 Subset and Its Association With Systemic Immunomodulatory Treatment in Non-infectious Uveitis
Source: Front Immunol. 2018 Oct 31;9:2519. doi: 10.3389/fimmu.2018.02519 (PMC6220365; doi:10.3389/fimmu.2018.02519)

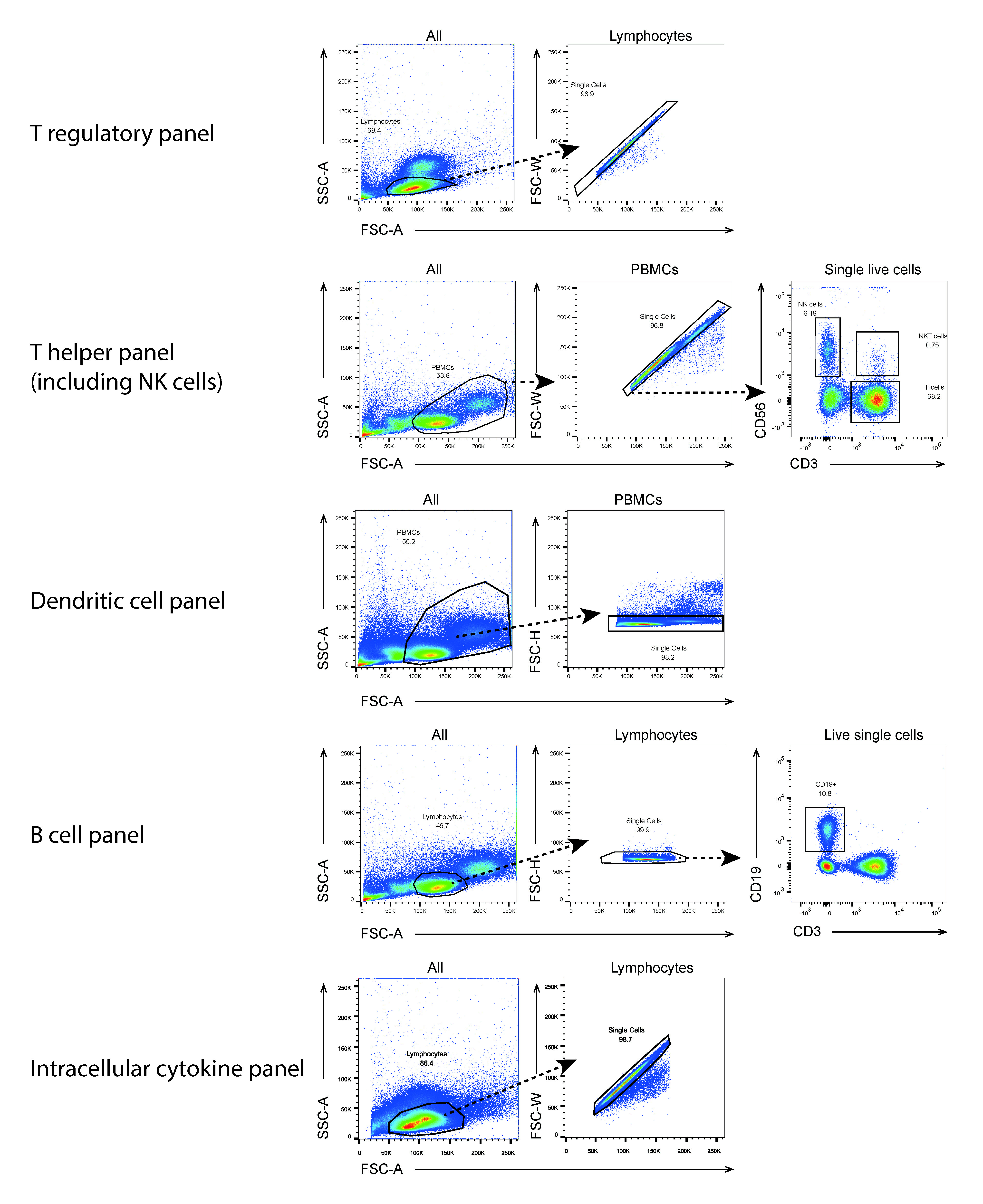

Supplement: Supplementary file 1 [file Image_1.JPEG]

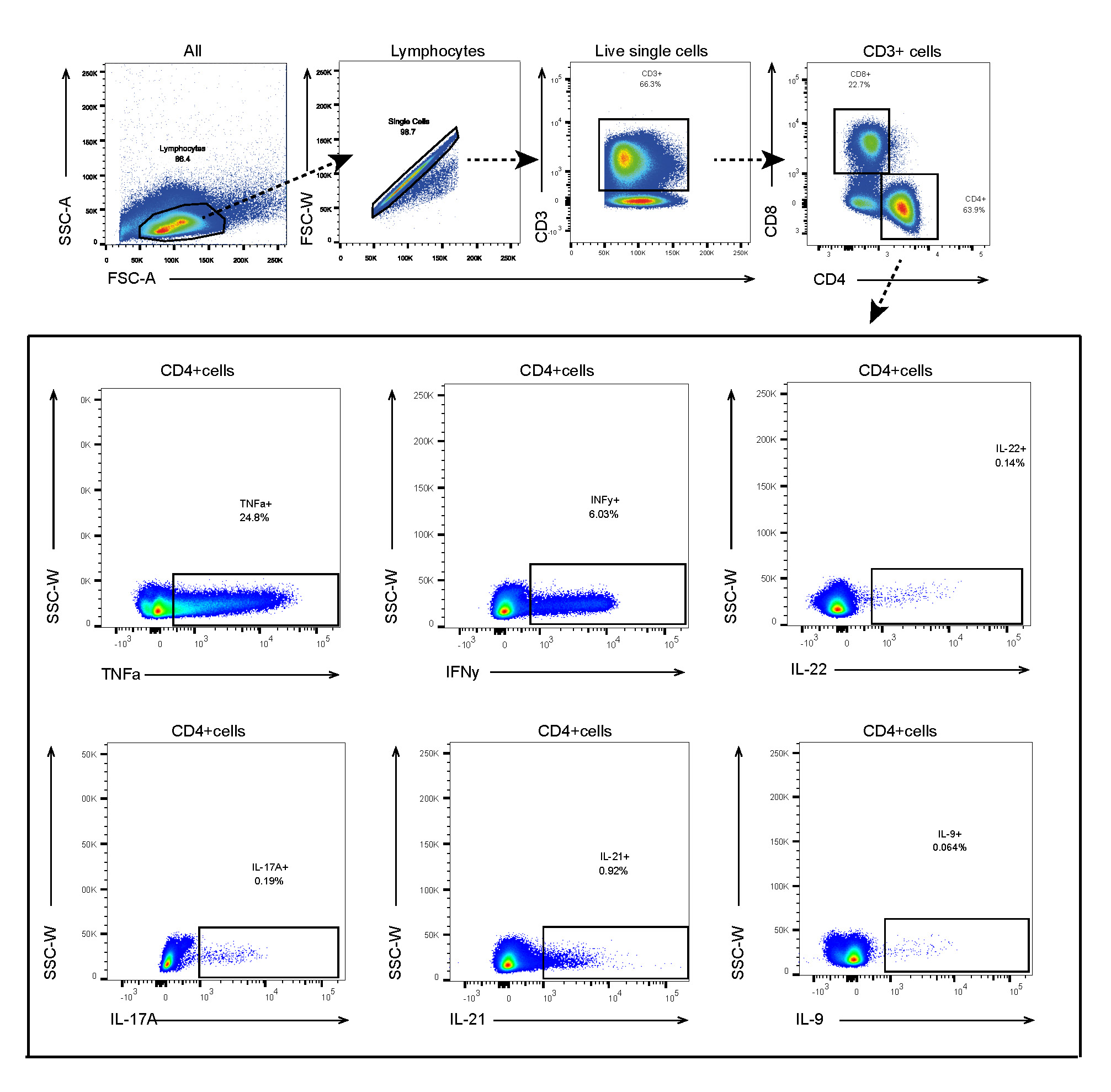

Supplement: Supplementary file 2 [file Image_2.JPEG]

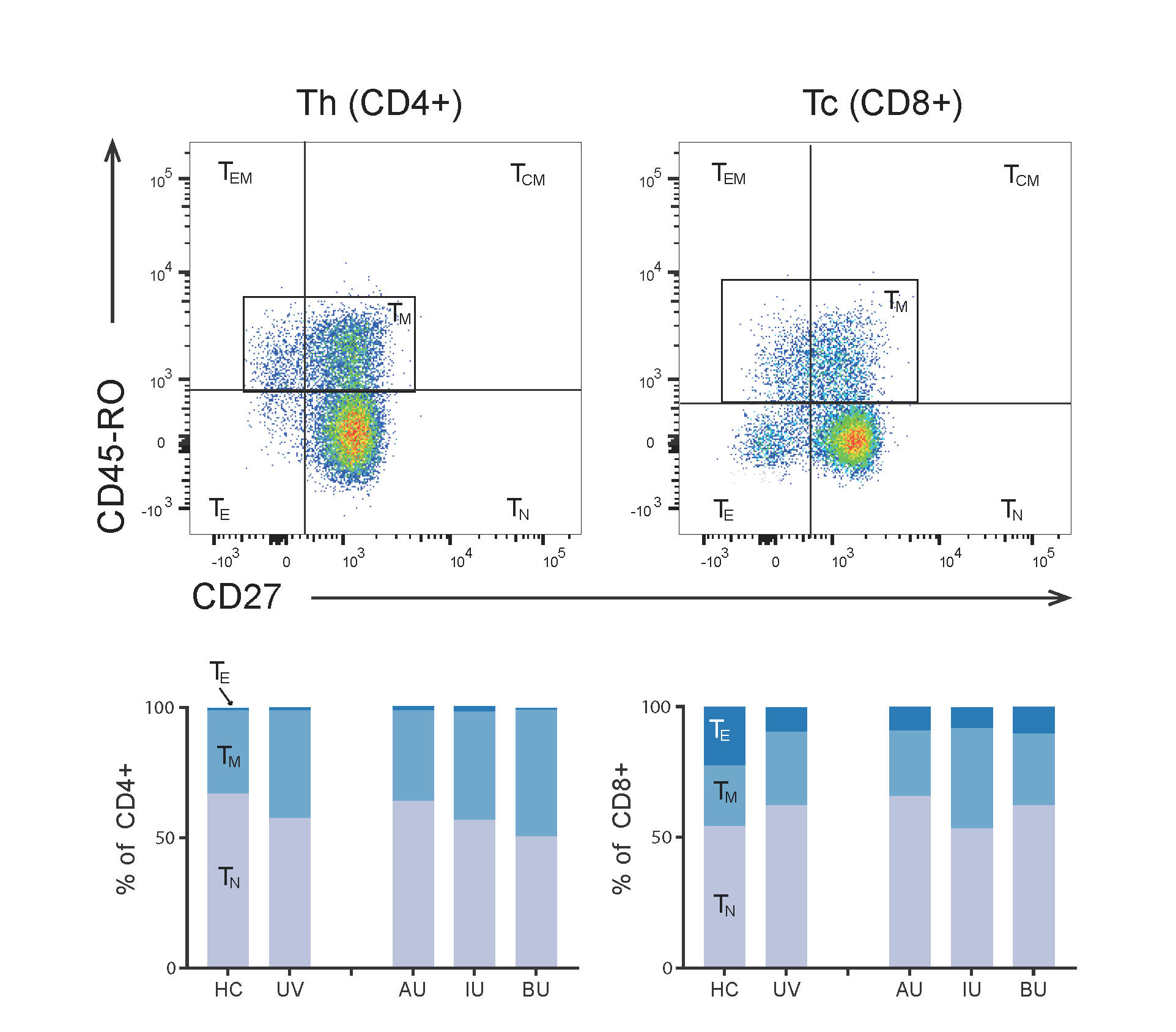

Supplement: Supplementary file 3 [file Image_3.JPEG]

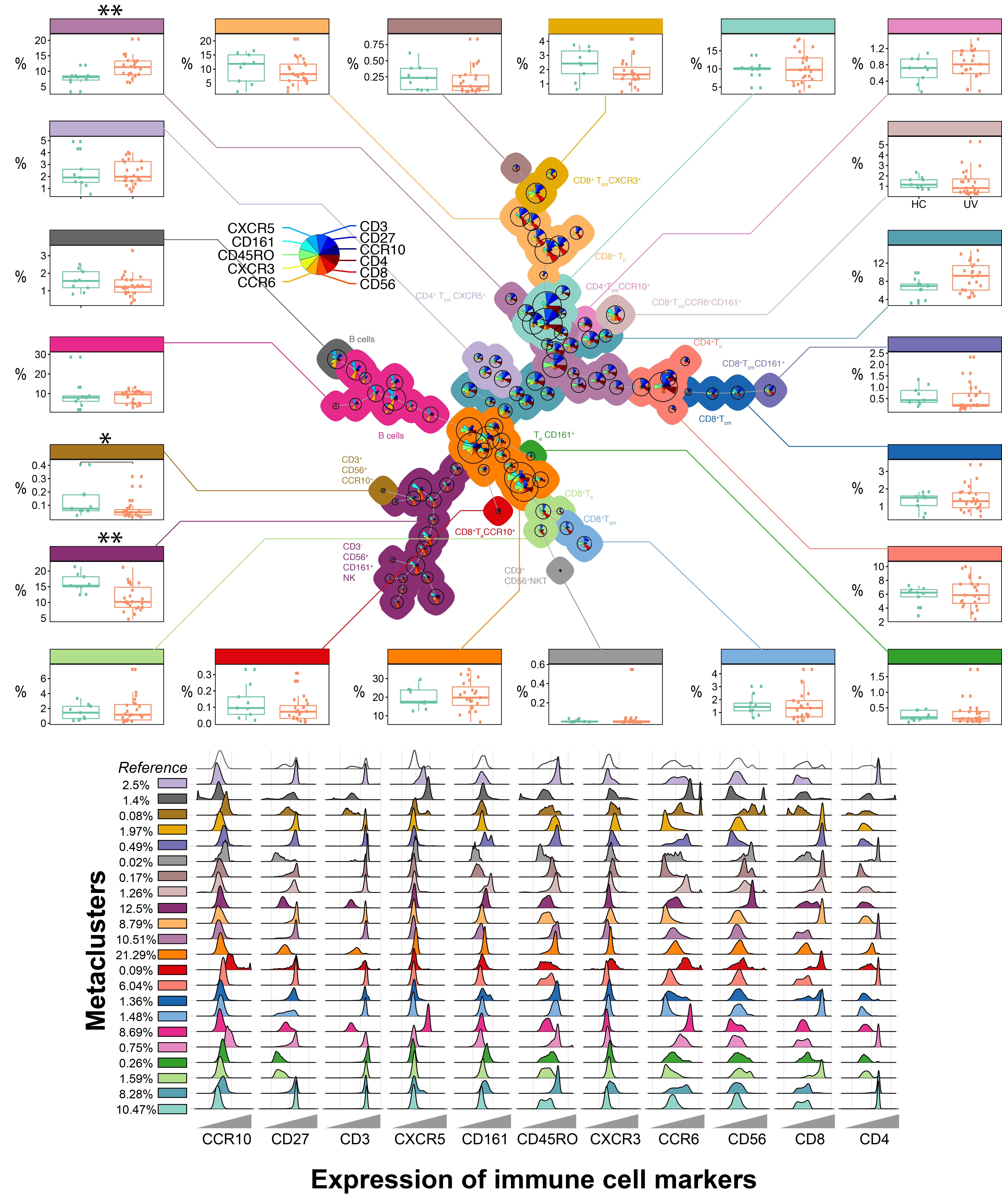

Supplement: Supplementary file 4 [file Image_4.JPEG]

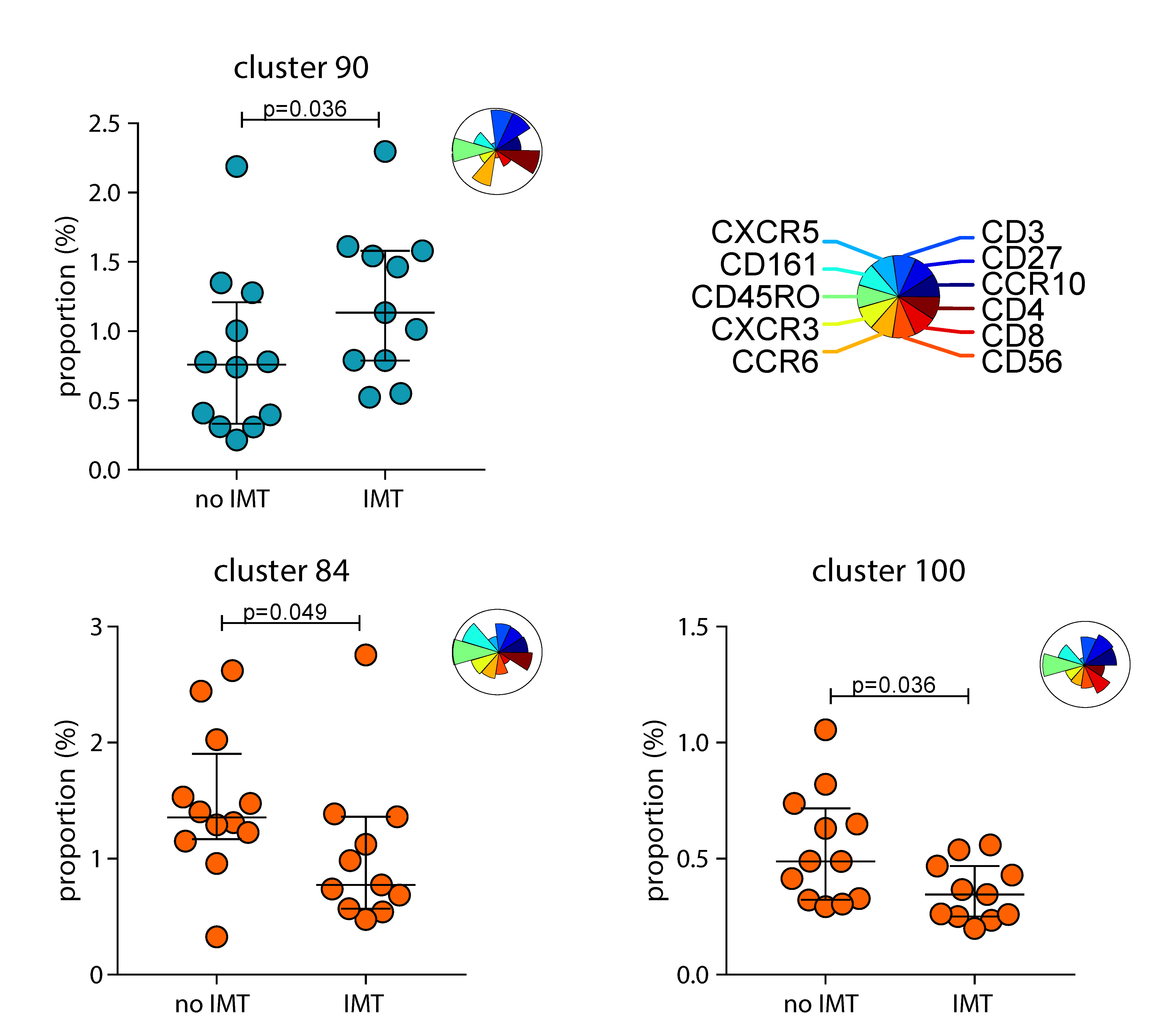

Supplement: Supplementary file 5 [file Image_5.JPEG]
