# Supplementary material for: High-Dimensional Profiling Reveals Heterogeneity of the Th17 Subset and Its Association With Systemic Immunomodulatory Treatment in Non-infectious Uveitis
Source: Front Immunol. 2018 Oct 31;9:2519. doi: 10.3389/fimmu.2018.02519 (PMC6220365; doi:10.3389/fimmu.2018.02519)
Supplement: Supplementary file 6 [file Table_1.docx]

**Supplementary Material**

**Supplementary Tables**

- **Table S1 -** Antibody panel used for Flow Cytometric staining of Peripheral Blood Mononuclear Cells
- **Table S2 -** Frequencies of manually gated cell subsets

**Supplementary Figures**

- **Supplementary Figure 1 -** Gating strategies to identify live single cells for all five panels
- **Figure S2 -** Gating strategy used for the intracellular cytokine panel
- **Figure S3 -** The proportions of naive and memory cells in Th (CD4^+^) and Tc (CD8^+^) cells
- **Figure S4 -** Details from unsupervised automatic gating using FlowSOM
- **Figure S5 -** Clusters identified by FlowSOM differentiating between patients that need prolonged systemic IMT after inclusion

**Supplementary Table 1** - **Antibody panel used for Flow Cytometric staining of Peripheral Blood Mononuclear Cells**

| Panel | Fluorochrome | Marker | Dilution | Clone | Company |
| --- | --- | --- | --- | --- | --- |
| **Dendritic** | FITC | CD123 | 1:20 | 7G3 | BD Pharmingen |
| **cell panel** | PerCP-CY5.5 | - | - | - | - |
|  | APC | CD1c | 1:10 | AD5-8E7 | Miltenyi |
|  | AF700 | CD3 | 1:40 | UCHT1 | BioLegend |
|  | AF700 | CD19 | 1:50 | HIB19 | eBioscience |
|  | AF700 | CD56 | 1:50 | B159 | BD Pharmingen |
|  | eFluor780 | CD14 | 1:40 | 61D3 | eBioscience |
|  | BV421 | HLA-DR | 1:100 | L243 | BioLegend |
|  | BV510 | CD16 | 1:100 | 3G8 | BD Horizon |
|  | BV605 | - | - | - | - |
|  | BV711 | CD141 | 1:50 | 1A4 | BD Horizon |
|  | PE | CD169 | 1:20 | 7-239 | eBioscience |
|  | PE-Cy7 | CD303 | 1:40 | 201A | BioLegend |
|  | PE-CF594 | CD11c | 1:200 | B-ly6 | BD Horizon |
| **Thelper** | FITC | CXCR3 | 1:40 | G025H7 | BioLegend |
| **panel** | PerCP-CY5.5 | CD4 | 1:25 | SK3 | BD |
|  | APC | CCR10 | 1:10 | 314305 | R&D Systems |
|  | AF700 | CD3 | 1:40 | UCHT1 | BioLegend |
|  | eFluor780 | CD27 | 1:20 | L128 | eBioscience |
|  | BV421 | CXCR5 | 1:40 | RF8B2 | BD |
|  | BV510 | CD161 | 1:20 | DX12 | BD Horizon |
|  | BV605 | CCR4 | 1:40 | 1G1 | BD Horizon |
|  | BV711 | CD45RO | 1:100 | UCHL1 | BioLegend |
|  | PE | CCR6 | 1:40 | 11A9 | BD Pharmingen |
|  | PE-Cy7 | CD8 | 1:200 | SK1 | BD |
|  | PE-CF594 | CD56 | 1:20 | B159 | BD |
| **Tregulatory** | FITC | - |  | - | - |
| **panel** | PerCP-CY5.5 | CD4 | 1:25 | SK3 | BD |
|  | APC | FoxP3 | 1:25 | PCH101 | eBioscience |
|  | AF700 | CD3 | 1:40 | UCHT1 | BioLegend |
|  | eFluor780 | - | - | - | - |
|  | BV421 | CD127 | 1:40 | HIL-7R-M21 | BD Horizon |
|  | BV510 | - | - | - | - |
|  | BV605 | - | - | - | - |
|  | BV711 | CD45RO | 1:100 | UCHL1 | BioLegend |
|  | PE | CD25 | 1:50 | 2A3 | BD |
|  | PE-Cy7 | CD8 | 1:200 | SK1 | BD |
|  | PE-CF594 | - | - | - | - |
| **B cel** | FITC | IgG | 1:500 | NA | Southern Biotech |
| **panel** | PerCP-CY5.5 | CD38 | 1:100 | HIT2 | BD Pharmingen |
|  | APC | CD27 | 1:50 | L128 | BD |
|  | AF700 | CD3 | 1:40 | UCHT1 | BioLegend |
|  | eFluor780 | CD19 | 1:20 | HIB19 | eBioscience |
|  | BV421 | IgM | 1:50 | G20-127 | BD |
|  | BV510 | IgD | 1:500 | IA6-2 | Biolegend |
|  | BV605 | - | - | - | - |
|  | BV711 | CD21 | 1:20 | B-ly4 | BD |
|  | PE | IgA | 1:5000 | NA | Southern Biotech |
|  | PE-Cy7 | CD10 | 1:100 | HI10A | BD |
|  | PE-CF594 | CD24 | 1:200 | ML5 | BD Horizon |
| **Intracellular** | FITC | IL-17A | 1:40 | eBio64DEC17 | eBioscience |
| **cytokine** | PerCP-CY5.5 | IFN-γ | 1:40 | 4S.B3 | eBioscience |
| **panel** | APC | IL-22 | 1:40 | IL22JOP | eBioscience |
|  | AF700 | CD3 | 1:40 | UCHT1 | BioLegend |
|  | eFluor780 | CD4 | 1:40 | RPA-T4 | eBioscience |
|  | BV421 | TNF-α | 1:100 | MAb11 | BD Horizon |
|  | BV510 | CD27 | 1:50 | L128 | BD Horizon |
|  | BV605 | - |  | - | - |
|  | BV711 | CD45RO | 1:100 | UCHL1 | BioLegend |
|  | PE | IL-21 | 1:10 | 3A3-N2.1 | BD Pharmingen |
|  | PE-Cy7 | CD8 | 1:200 | SK1 | BD |
|  | PE-CF594 | IL-9 | 1:20 | MH9A3 | BD Horizon |

**Abbreviations:** IFN: interferon, IL: interleukin, NA: not applicable, Treg: T regulatory cell, TNF: tumor necrosis factor

**Supplementary Table 2 - Frequencies of manually gated cell subsets**

| Cell subset | % of | N | HC | UV |  | AU | IU | BU |  | posthoc | |
| --- | --- | --- | --- | --- | --- | --- | --- | --- | --- | --- | --- |
|  |  |  | **median (IQR)** | **median (IQR)** | **p-val.** | **median (IQR)** | **median (IQR)** | **median (IQR)** | **p-val.** | **group** | **p-adj*** |
| PBMCs (live single) | all cells | 42 | 51.8 (46.7-60.7) | 61.1 (54.1-68.0) | **0.023** | 60.9 (52.5-68.3) | 61.2 (54.4-69.9) | 61.6 (53.3-69.9) | 0.16 |  |  |
| NK (CD3^-^CD56^+^) | PBMCs | 32 | 14.4 (11.8-16.3) | 9.6 (7.4-12.8) | **0.005** | 9.7 (7.5-12.0) | 10.5 (8.2-14.8) | 7.4 (6.2-12.8) | **0.02** | **HC-BU** | **0.04** |
| NKT (CD3^+^CD56^+^) | PBMCs | 32 | 1.00 (0.6-2.6) | 1.0 (0.6-2.6) | 0.95 | 1.0 (0.6-1.7) | 1.1 (0.5-2.2) | 0.9 (0.5-5.0) | 0.97 |  |  |
| Tcells (CD3^+^CD56^-^) | PBMCs | 42 | 55.9 (52.4-62.5) | 55.3 (49.3-65.1) | 0.69 | 59.1 (50.0-66.3) | 52.8 (47.0-58.5) | 59.4 (49.5-65.6) | 0.46 |  |  |
| CD4^+^ | Tcells | 42 | 57.6 (51.1-70.5) | 64.8 (56.5-73.6) | 0.09 | 64.8 (55.3-73.3) | 64.8 (53.2-71.7) | 64.9 (59.4-77.8) | 0.28 |  |  |
| T_N_ (CD45RO^-^CD27^+^) | Th (CD4+) | 42 | 65.9 (60.6-69.6) | 56.0 (46.3-62.4) | **0.004** | 62.0 (54.1-71.7) | 55.4 (43.9-62.4) | 49.3 (39.7-56.9) | **0.001** | **HC-BU** | **0.003** |
|  |  |  |  |  |  |  |  |  |  | **AU-BU** | **0.04** |
| T_E_ (CD45RO^-^CD27^-^ ) | Th (CD4+) | 42 | 1.1 (0.7-1.5) | 0.9 (0.7-1.7) | 0.96 | 0.9 (0.6-1.5) | 1.5 (0.8-2.7) | 0.8 (0.6-2.0) | 0.30 |  |  |
| T_M_ (CD45RO^+^) | Th (CD4+) | 42 | 31.1 (25.7-36.8) | 40.2 (33.8-49.3) | **0.008** | 33.5 (25.2-42.0) | 40.2 (33.8-49.6) | 47.1 (38.8-52.8) | **0.001** | **HC-BU** | **0.002** |
|  |  |  |  |  |  |  |  |  |  | **AU-BU** | **0.01** |
| Th1 (CCR6^-^CXCR3^+^) | CD4 T_M_ | 32 | 16.3 (9.0-23.7) | 9.9 (8.1-13.8) | **0.044** | 9.5 (6.2-15.1) | 9.5 (8.3-13.5) | 9.9 (8.1-14.5) | 0.22 |  |  |
| Th1 (CCR6^-^CXCR3^+^CD45RO^+^) | Th (CD4+) | 32 | 5.1 (3.0-6.6) | 3.8 (2.5-5.3) | 0.44 | 2.5 (2.1-4.2) | 4.3 (2.8-4.8) | 5.3 (3.4-7.0) | 0.16 |  |  |
| Th2 (CCR6^-^CXCR3^-^) | CD4 T_M_ | 32 | 41.7 (37.6-54.0) | 46.8 (41.8-55.5) | 0.40 | 52.0 (47.1-55.4) | 43.1 (41.9-64.8) | 39.6 (31.4-50.9) | 0.17 |  |  |
| Th2 (CCR6^-^CXCR3^-^CD45RO^+^) | Th (CD4+) | 32 | 12.7 (10.5-17.5) | 18.6 (13.6-21.4) | **0.049** | 16.6 (11.8-20.8) | 19.9 (15.8-24.3) | 16.5 (14.6-32.0) | 0.15 |  |  |
| Th17 (CCR6^+^CXCR3^-^) | CD4 T_M_ | 32 | 25.7 (23.7-26.6) | 31.3 (28.0-36.5) | **0.018** | 29.7 (28.8-34.8) | 31.1 (21.3-37.9) | 33.9 (30.2-37.9) | 0.07 |  |  |
| Th17 (CCR6^+^CXCR3^-^CD45RO^+^) | Th (CD4+) | 32 | 8.0 (6.4-9.2) | 12.4 (7.5-15.2) | **0.038** | 9.1 (6.5-13.5) | 12.1 (7.4-17.0) | 14.4 (13.4-19.6) | **0.010** | **HC-BU** | **0.009** |
| Th17.1 (CCR6^+^CXCR3^+^) | CD4 T_M_ | 32 | 10.0 (7.0-14.6) | 8.2 (5.4-13.7) | 0.52 | 7.2 (5.1-9.3) | 9.3 (5.1-13.1) | 16.5 (5.9-17.6) | 0.31 |  |  |
| Th17.1 (CCR6^+^CXCR3^+^CD45RO^+^) | Th (CD4+) | 32 | 2.8 (2.1-4.5) | 3.5 (2.0-6.0) | 0.82 | 2.1 (1.3-3.3) | 3.8 (1.7-5.6) | 6.4 (2.1-9.8) | 0.06 |  |  |
| Th22 (CCR6^+^CCR10^+^) | CD4 T_M_ | 32 | 1.7 (1.1-2.5) | 1.9 (1.1-3.1) | 0.93 | 2.3 (1.6-3.2) | 1.3 (0.7-2.3) | 1.8 (1.1-5.3) | 0.57 |  |  |
| Th22 (CCR6^+^CCR10^+^CD45RO^+^) | Th (CD4+) | 32 | 0.5 (0.3-0.9) | 0.8 (0.4-1.2) | 0.54 | 0.9 (0.4-1.2) | 0.5 (0.3-1.1) | 1.2 (0.4-2.1) | 0.36 |  |  |
| Tfh (CXCR5^+^CD27^+^) | CD4 T_M_ | 42 | 25.0 (17.4-29.7) | 17.0 (14.1-22.2) | **0.002** | 16.1 (13.8-22.9) | 14.8 (13.2-18.5) | 19.2 (16.2-23.7) | **0.011** | **HC-IU** | **0.01** |
| Tfh (CXCR5^+^CD27^+^CD45RO^+^) | Th (CD4+) | 42 | 7.8 (5.0-10.0) | 7.0 (5.4-8.5) | 0.51 | 6.4 (3.6-7.1) | 6.5 (5.1-7.4) | 9.0 (8.0-10.3) | **0.033** | **-** |  |
| CCR6^+^ | CD4 T_M_ | 32 | 35.9 (34.9-41.1) | 40.1 (36.8-47.5) | 0.20 | 38.0 (36.9-40.1) | 42.1 (26.7-49.3) | 47.5 (40.1-53.3) | 0.18 |  |  |
| CCR6^+^CD45RO^+^ | Th (CD4+) | 32 | 10.4 (9.3-14.5) | 15.7 (10.0-20.7) | 0.10 | 11.5 (8.3-15.8) | 16.0 (9.2-21.4) | 20.7 (15.7-27.1) | **0.009** | **HC-BU** | **0.02** |
|  |  |  |  |  |  |  |  |  |  | **AU-BU** | **0.02** |
| CD161^+^ | CD4 T_M_ | 42 | 22.3 (16.7-25.5) | 19.9 (15.7-23.9) | 0.41 | 17.1 (13.7-21.7) | 21.7 (15.9-23.6) | 18.8 (15.3-27.3) | 0.48 |  |  |
| CD161^+^CD45RO^+^ | Th (CD4+) | 42 | 5.7 (5.2-8.0) | 7.8 (6.0-10.4) | 0.17 | 6.0 (3.8-7.9) | 9.8 (6.0-10.9) | 9.2 (7.6-11.3) | **0.022** | **AU-BU** | **0.04** |
| CD161^+^CCR6^+^ | CD4 T_M_ | 32 | 10.3 (6.4-14.2) | 8.2 (5.5-12.9) | 0.46 | 8.2 (6.5-9.7) | 9.7 (4.0-14.4) | 9.9 (5.5-15.4) | 0.73 |  |  |
| CD161^+^CCR6^+^CD45RO^+^ | Th (CD4+) | 32 | 3.0 (2.2-3.9) | 2.9 (2.0-6.0) | 0.71 | 2.6 (1.9-3.0) | 3.9 (1.5-7.0) | 3.8 (2.9-7.4) | 0.23 |  |  |
| CD161^-^CCR6^+^ | CD4 T_M_ | 32 | 28.1 (20.4-30.5) | 30.6 (26.4-34.6) | 0.22 | 28.4 (26.8-31.3) | 28.5 (18.3-35.7) | 34.6 (31.6-41.6) | 0.08 |  |  |
| CD161^-^CCR6^+^CD45RO^+^ | Th (CD4+) | 32 | 6.7 (6.1-11.2) | 12.1 (7.1-15.3) | 0.08 | 9.0 (6.3-11.8) | 10.9 (6.3-14.5) | 16.3 (12.9-21.4) | **0.012** | **HC-BU** | **0.01** |
|  |  |  |  |  |  |  |  |  |  | **AU-BU** | **<0.05** |
| CD161^+^ Th17 (CCR6+CXCR3-) | Th17 cells | 32 | 31.4 (20.9-37.4) | 29.4 (22.2-33.6) | 0.54 | 28.5 (22.8-32.2) | 32.8 (20.4-36.3) | 25.7 (21.1-36.1) | 0.87 |  |  |
| CD161^+^ Th17 (CCR6+CXCR3-) | CD4 T_M_ | 32 | 8.1 (6.1-10.2) | 8.6 (7.0-12.2) | 0.54 | 8.0 (7.3-9.4) | 9.3 (5.3-12.6) | 8.8 (7.0-13.4) | 0.88 |  |  |
| CD161^+^ Th17 (CCR6+CXCR3-) | Th (CD4+) | 32 | 2.4 (1.9-2.9) | 3.4 (2.1-4.7) | 0.06 | 2.4 (1.8-3.4) | 3.5 (2.1-5.6) | 4.5 (4.1-5.0) | **0.033** | **HC-BU** | **0.04** |
| CD161^-^ Th17 (CCR6+CXCR3-) | Th17 cells | 32 | 68.2 (61.9-79.1) | 73.1 (66.2-77.0) | 0.41 | 73.5 (67.8-76.7) | 67.9 (64.5-79.6) | 74.1 (63.8-80.3) | 0.85 |  |  |
| CD161^-^ Th17 (CCR6+CXCR3-) | CD4 T_M_ | 32 | 16.4 (15.1-20.0) | 22.4 (19.8-26.4) | **0.014** | 22.0 (21.1-23.8) | 20.3 (15.6-26.2) | 24.2 (22.4-27.3) | **0.035** | **HC-BU** | **0.03** |
| CD161^-^ Th17 (CCR6+CXCR3-) | Th (CD4+) | 32 | 5.6 (4.0-7.2) | 9.0 (5.6-11.8) | **0.029** | 6.5 (4.6-9.4) | 7.6 (5.7-12.0) | 10.2 (9.0-14.8) | **0.014** | **HC-BU** | **0.01** |
| CD161^+^CCR6^-^ | CD4 T_M_ | 32 | 4.3 (2.3-6.0) | 2.6 (1.8-4.0) | 0.10 | 2.1 (1.5-3.6) | 2.9 (2.0-6.8) | 2.9 (1.0-4.0) | 0.24 |  |  |
| CD161^+^CCR6^-^CD45RO^+^ | Th (CD4+) | 32 | 1.1 (0.7-1.7) | 1.0 (0.6-1.8) | 0.65 | 0.6 (0.4-1.0) | 1.3 (0.7-2.7) | 1.4 (0.6-2.0) | 0.27 |  |  |
| Treg (FoxP3^+^CD25^hi^) | CD4 T_M_ | 36 | 9.1 (6.6-12.4) | 9.4 (6.8-11.1) | 0.86 | 9.6 (8.6-12.1) | 8.5 (6.1-11.1) | 9.4 (5.6-12.1) | 0.67 |  |  |
| Treg (FoxP3^+^CD25^hi^) | Th (CD4+) | 36 | 5.5 (3.4-5.9) | 5.3 (4.5-6.2) | 0.48 | 4.8 (4.4-6.1) | 5.2 (4.3-6.5) | 5.8 (4.3-7.9) | 0.86 |  |  |
| CD8+ | Tcells (CD3^+^) | 42 | 35.1 (23.6-40.7) | 26.9 (19.3-35.2) | **0.027** | 25.3 (16.6-35.9) | 26.9 (22.3-35.9) | 27.2 (18.2-32.8) | 0.15 |  |  |
| T_N_ (CD45RO^-^CD27^+^) | Tc (CD8+) | 42 | 49.0 (31.5-63.4) | 58.8 (35.5-73.9) | 0.30 | 61.5 (36.1-81.8) | 44.1 (35.5-73.9) | 59.0 (28.8-64.4) | 0.61 |  |  |
| T_E_ (CD45RO^-^CD27^-^) | Tc (CD8+) | 42 | 20.3 (14.7-29.0) | 9.2 (3.5-21.9) | **0.029** | 8.5 (3.2-24.3) | 6.8 (3.1-20.8) | 9.9 (4.1-43.4) | 0.12 |  |  |
| T_M_ (CD45RO^+^) | Tc (CD8+) | 42 | 20.8 (16.0-36.5) | 26.4 (19.5-32.8) | 0.77 | 23.3 (11.7-31.0) | 31.9 (19.9-38.8) | 26.0 (22.6-27.6) | 0.52 |  |  |
| CXCR3^-^CCR6^+^ | CD8 T_M_ | 32 | 13.1 (6.9-21.4) | 9.8 (6.4-15.3) | 0.69 | 13.4 (8.9-16.8) | 8.8 (6.4-30.1) | 6.4 (5.4-15.3) | 0.78 |  |  |
| CXCR3^+^CCR6^+^ | CD8 T_M_ | 32 | 4.7 (2.5-6.2) | 3.9 (1.4-5.8) | 0.30 | 3.9 (1.2-5.8) | 4.1 (1.7-5.8) | 2.3 (1.9-5.8) | 0.77 |  |  |
| CXCR3^+^CCR6^-^ | CD8 T_M_ | 32 | 20.4 (15.0-26.5) | 16.4 (10.5-24.6) | 0.23 | 16.8 (7.1-23.7) | 15.1 (8.7-24.4) | 14.3 (13.7-27.6) | 0.66 |  |  |
| CXCR3^-^CCR6^-^ | CD8 T_M_ | 32 | 58.3 (48.5-71.8) | 68.3 (45.6-80.7) | 0.41 | 68.2 (55.9-79.9) | 67.2 (42.2-83.1) | 68.3 (45.6-79.0) | 0.86 |  |  |
| CD161^+^CCR6^+^ | CD8 T_M_ | 32 | 10.8 (4.8-20.5) | 7.0 (3.1-13.8) | 0.41 | 9.1 (5.2-13.6) | 5.7 (1.0-27.1) | 3.1 (1.6-13.8) | 0.63 |  |  |
| CD161^-^CCR6^+^ | CD8 T_M_ | 32 | 6.0 (4.9-7.9) | 6.8 (5.4-8.1) | 0.65 | 6.2 (4.9-8.3) | 6.8 (6.1-8.1) | 6.7 (4.5-8.0) | 0.92 |  |  |
| CCR10^+^ | CD8 T_M_ | 42 | 1.2 (0.8-2.3) | 1.6 (1.0-2.8) | 0.45 | 1.9 (1.1-5.2) | 0.9 (0.7-1.3) | 2.3 (1.6-3.9) | **0.011** | **IU-BU** | **0.01** |
| CXCR3^-^CCR6^+^ | CD8 T_E_ | 32 | 4.1 (2.2-6.7) | 4.3 (2.4-7.3) | 0.79 | 3.1 (2.5-7.2) | 4.9 (4.3-12.5) | 2.2 (1.8-14.9) | 0.33 |  |  |
| CXCR3^+^CCR6^+^ | CD8 T_E_ | 32 | 1.5 (0.6-2.9) | 1.0 (0.3-1.6) | 0.16 | 0.3 (0.2-2.2) | 1.3 (0.8-1.6) | 0.8 (0.3-1.3) | 0.39 |  |  |
| CXCR3^+^CCR6^-^ | CD8 T_E_ | 32 | 4.1 (1.9-9.9) | 3.0 (1.3-4.3) | 0.15 | 1.7 (0.7-6.3) | 2.8 (1.6-6.5) | 3.3 (2.9-4.3) | 0.40 |  |  |
| CXCR3^-^CCR6^-^ | CD8 T_E_ | 32 | 91.1 (83.3-93.1) | 92.5 (83.3-95.7) | 0.43 | 94.7 (84.7-97.0) | 90.5 (78.8-92.9) | 93.5 (80.6-96.1) | 0.34 |  |  |
| CD161^+^CCR6^+^ | CD8 T_E_ | 32 | 1.4 (1.0-2.1) | 0.9 (0.3-2.7) | 0.57 | 1.2 (0.6-1.9) | 2.0 (0.1-7.3) | 0.4 (0.2-6.8) | 0.74 |  |  |
| CD161^-^CCR6^+^ | CD8 T_E_ | 32 | 3.6 (2.8-4.6) | 3.4 (2.1-5.4) | 0.95 | 2.9 (1.6-5.2) | 5.2 (3.2-5.5) | 3.3 (1.2-3.4) | 0.36 |  |  |
| CCR10^+^ | CD8 T_E_ | 42 | 0.4 (0.3-1.0) | 0.5 (0.1-0.8) | 0.84 | 0.4 (0.2-1.0) | 0.3 (0.1-0.6) | 0.6 (0.1-1.2) | 0.57 |  |  |
| IL9^+^ | Th (CD4+) | 42 | 0.03 (0.02-0.05) | 0.04 (0.02-0.06) | 0.64 | 0.04 (0.02-0.06) | 0.02 (0.01-0.06) | 0.04 (0.02-0.07) | 0.75 |  |  |
| IL17^+^ | Th (CD4+) | 42 | 0.28 (0.19-0.37) | 0.29 (0.19-0.46) |  | 0.29 (0.25-0.31) | 0.20 (0.14-0.40) | 0.46 (0.22-0.93) | 0.16 |  |  |
| IL21^+^ | Th (CD4+) | 42 | 1.9 (1.1-2.9) | 1.6 (1.4-2.3) | 0.69 | 1.7 (1.1-3.2) | 1.6 (1.2-2.0) | 1.6 (1.5-2.4) | 0.86 |  |  |
| IL22^+^ | Th (CD4+) | 42 | 0.18 (0.09-0.24) | 0.19 (0.09-0.36) |  | 0.19 (0.10-0.28) | 0.14 (0.08-0.38) | 0.38 (0.11-1.00) | 0.26 |  |  |
| TNFa^-^ IFN^+^ | Th (CD4+) | 42 | 1.3 (0.5-2.1) | 1.4 (1.1-2.0) | 0.34 | 1.2 (0.9-1.8) | 1.4 (1.0-2.4) | 1.5 (1.2-2.5) | 0.61 |  |  |
| TNFa^+^ IFN^+^ | Th (CD4+) | 42 | 5.3 (2.9-9.9) | 7.3 (4.5-11.0) | 0.23 | 6.2 (3.6-9.5) | 5.5 (4.1-13.3) | 9.0 (7.1-14.5) | 0.20 |  |  |
| TNFa^+^ IFN^-^ | Th (CD4+) | 42 | 29.4 (21.4-34.8) | 27.5 (20.4-35.7) | 0.92 | 25.3 (17.1-34.9) | 20.5 (15.3-32.1) | 33.4 (25.4-39.0) | 0.25 |  |  |
| TNFa^-^ IFN^-^ | Th (CD4+) | 42 | 65.5 (51.4-74.8) | 61.0 (49.2-73.4) | 0.69 | 61.8 (56.6-78.3) | 70.2 (52.1-75.0) | 54.1 (46.9-61.9) | 0.18 |  |  |
| IL9^+^ | Tc (CD8+) | 42 | 0.52 (0.44-0.74) | 0.62 (0.42-0.92) | 0.49 | 0.58 (0.40-0.74) | 0.59 (0.27-0.84) | 0.92 (0.57-1.06) | 0.22 |  |  |
| IL17^+^ | Tc (CD8+) | 42 | 0.08 (0.05-0.15) | 0.06 (0.04-0.09) |  | 0.06 (0.05-0.10) | 0.06 (0.03-0.10) | 0.06 (0.05-0.09) | 0.31 |  |  |
| IL21^+^ | Tc (CD8+) | 42 | 0.01 (0.00-0.02) | 0.01 (0.01-0.02) | 0.33 | 0.01 (0.01-0.02) | 0.01 (0.01-0.02) | 0.02 (0.01-0.04) | 0.13 |  |  |
| IL22^+^ | Tc (CD8+) | 42 | 0.02 (0.01-0.04) | 0.02 (0.01-0.08) |  | 0.02 (0.01-0.04) | 0.02 (0.01-0.04) | 0.05 (0.01-0.14) | 0.55 |  |  |
| TNFa^-^ IFN^+^ | Tc (CD8+) | 42 | 11.8 (6.3-16.8) | 10.8 (4.7-15.4) | 0.63 | 5.1 (4.2-14.5) | 10.7 (5.1-17.0) | 12.8 (6.2-16.3) | 0.68 |  |  |
| TNFa^+^ IFN^+^ | Tc (CD8+) | 42 | 24.8 (12.8-34.7) | 22.7 (11.6-34.4) | 0.77 | 20.6 (7.7-35.3) | 20.6 (9.4-29.3) | 24.5 (18.2-40.2) | 0.55 |  |  |
| TNFa^+^ IFN^-^ | Tc (CD8+) | 42 | 6.7 (4.4-9.4) | 7.3 (4.7-12.4) | 0.54 | 6.5 (4.3-9.2) | 9.0 (3.2-14.1) | 6.9 (5.0-13.8) | 0.64 |  |  |
| TNFa^-^ IFN^-^ | Tc (CD8+) | 42 | 56.0 (41.0-72.0) | 51.1 (43.8-71.2) | 0.75 | 53.9 (38.8-83.8) | 53.2 (45.8-73.9) | 50.4 (41.3-57.8) | 0.57 |  |  |
| HLA-DR^+^Lin^-^ | PBMCs | 45 | 14.9 (11.0-20.5) | 17.9 (12.6-24.3) | 0.32 | 16.1 (12.1-22.5) | 23.5 (18.0-27.8) | 13.7 (12.4-23.2) | 0.19 |  |  |
| Classical monocytes (CD14^+^CD16^-^) | HLA-DR^+^Lin^-^ | 45 | 75.1 (68.9-77.3) | 76.4 (74.1-80.4) | 0.20 | 76.8 (74.8-79.8) | 76.6 (75.3-83.5) | 73.4 (68.0-78.6) | 0.14 |  |  |
| Classical monocytes (CD14^+^CD16^-^HLA-DR^+^Lin^-^) | PBMCs | 45 | 11.5 (8.3-15.0) | 12.5 (9.3-19.3) | 0.32 | 12.5 (9.7-17.5) | 18.4 (13.9-21.9) | 9.4 (8.5-19.8) | 0.12 |  |  |
| Intermediate monocytes (CD14^+^CD16^+^) | HLA-DR^+^Lin^-^ | 45 | 3.8 (3.5-5.7) | 4.1 (2.8-5.6) | 0.72 | 3.9 (2.7-4.9) | 2.9 (2.7-4.7) | 5.2 (3.9-7.0) | 0.33 |  |  |
| Intermediate Monocytes (CD16^+^CD14^+^HLA-DR^+^Lin^-^) | PBMCs | 45 | 0.6 (0.5-1.0) | 0.7 (0.5-1.0) | 0.57 | 0.6 (0.4-0.8) | 0.8 (0.6-1.1) | 0.6 (0.5-1.1) | 0.57 |  |  |
| Non-classical monocytes (CD16^+^CD14^-^) | HLA-DR^+^Lin^-^ | 45 | 5.9 (4.8-11.8) | 5.7 (2.2-8.3) | 0.06 | 5.9 (2.3-7.6) | 3.7 (1.1-7.1) | 7.9 (2.9-10.5) | 0.11 |  |  |
| Non-classical monocytes (CD16^+^CD14^-^HLA-DR^+^Lin^-^) | PBMCs | 45 | 1.2 (0.6-2.1) | 0.9 (0.3-1.6) | 0.24 | 1.0 (0.2-1.8) | 0.5 (0.3-1.7) | 1.1 (0.4-1.4) | 0.69 |  |  |
| pDC (CD303^+^) | CD14^-^CD16^-^HLA-DR^+^Lin^-^ | 45 | 26.9 (14.4-28.8) | 12.1 (9.0-14.8) | **0.001** | 12.2 (9.0-15.5) | 11.9 (7.7-15.5) | 12.2 (10.5-14.3) | **0.013** | **-** |  |
| pDC (CD303^+^CD14^-^CD16^-^) | HLA-DR^+^Lin^-^ | 45 | 2.4 (1.7-3.2) | 1.2 (0.8-1.6) | **0.001** | 1.1 (0.8-1.5) | 1.1 (0.8-1.4) | 1.2 (0.7-1.9) | **0.010** | **HC-AU** | **<0.05** |
|  |  |  |  |  |  |  |  |  |  | **HC-IU** | **0.03** |
| pDC (CD303^+^CD14^-^CD16^-^HLA-DR^+^Lin^-^) | PBMCs | 45 | 0.3 (0.2-0.5) | 0.2 (0.1-0.3) | **0.035** | 0.2 (0.1-0.3) | 0.3 (0.1-0.4) | 0.2 (0.1-0.3) | 0.11 |  |  |
| mDC2 (CD141^+^) | CD14^-^CD16^-^HLA-DR^+^Lin^-^ | 45 | 1.8 (1.2-2.6) | 1.7 (0.9-2.2) | 0.41 | 1.6 (0.6-2.1) | 1.7 (0.9-2.2) | 1.8 (1.1-2.3) | 0.70 |  |  |
| mDC2 (CD141^+^) | HLA-DR^+^Lin^-^ | 45 | 0.2 (0.1-0.2) | 0.1 (0.1-0.2) | 0.19 | 0.1 (0.1-0.2) | 0.2 (0.1-0.2) | 0.2 (0.1-0.2) | 0.27 |  |  |
| mDC2 (CD141^+^) | PBMCs | 45 | 0.0 (0.0-0.0) | 0.0 (0.0-0.0) | 0.84 | 0.0 (0.0-0.0) | 0.0 (0.0-0.0) | 0.0 (0.0-0.0) | 0.45 |  |  |
| mDC1 (CD1^+^CD11c^+^) | CD14^-^CD16^-^HLA-DR^+^Lin^-^ | 45 | 9.6 (7.1-13.3) | 10.1 (6.9-17.3) | 0.89 | 8.1 (5.5-15.1 | 10.9 (7.6-14.7) | 13.1 (8.6-20.8) | 0.48 |  |  |
| mDC1 (CD1^+^CD11c^+^) | HLA-DR^+^Lin^-^ | 45 | 0.9 (0.7-1.5) | 1.0 (0.6-1.4) | 0.81 | 0.8 (0.5-1.3) | 0.9 (0.5-1.4) | 1.1 (0.9-1.9) | 0.34 |  |  |
| mDC1 (CD1^+^CD11c^+^) | PBMCs | 45 | 0.1 (0.1-0.2) | 0.2 (0.1-0.2) | 0.41 | 0.1 (0.1-0.3) | 0.2 (0.1-0.3) | 0.2 (0.1-0.2) | 0.38 |  |  |
| B cells (CD19^+^) | Lymphocytes | 31 | 8.9 (6.7-11.3) | 12.1 (9.6-14.4) | **0.03** | 11.1 (7.1-12.3) | 13.1 (9.4-16.7) | 12.2 (9.9-14.4) | 0.10 |  |  |
| Transitional B cells (CD24^+^CD38^+^CD10^+^) | Bcells | 31 | 6.2 (4.6-9.8) | 4.9 (2.6-7.2) | 0.09 | 5.2 (2.0-7.3) | 4.6 (3.2-5.5) | 4.6 (1.6-7.6) | 0.40 |  |  |
| Plasmablast (CD10^-^CD38^hi^CD27^hi^) | Bcells | 31 | 1.4 (0.5-2.1) | 0.6 (0.5-1.1) | 0.09 | 0.6 (0.4-1.1) | 0.6 (0.5-1.0) | 0.7 (0.5-1.1) | 0.38 |  |  |
| Bregs (CD28^hi^CD24^hi^) | Bcells | 31 | 6.5 (4.3-10.0) | 4.6 (2.4-7.5) | 0.12 | 3.6 (2.3-8.8) | 4.7 (3.4-5.3) | 4.6 (1.5-8.0) | 0.45 |  |  |

P-values between HC and UV are from Wilcoxon rank-sum test , p-values between uveitis subgroups are from Kruskal-Wallis (KW) test with post-hoc Dunn’s correction for multiple testing (*).

**Abbreviations:** Breg: regulatory B cell, AU: HLA-B27 associated anterior uveitis, BU: Birdshot uveitis, HC: healthy control, IFN: interferon, IL: interleukin, IQR: interquartile range, IU: idiopathic intermediate uveitis, mDC: myeloid dendritic cell, Tc: cytotoxic T cell, T_E_: effector T cell, Th: T helper cell, T_M_: memory T cell, T_N_: naïve T cell, Treg: T regulatory cell, PBMC: peripheral blood mononuclear cell, pDC: plasmacytoid dendritic cell, TNF: tumor necrosis factor, UV: all uveitis patients combined.

**FIGURE LEGENDS** - supplementary Figures

**Supplementary Figure 1 | Gating strategies to identify live single cells for all five panels.** Live single cells are identified using stringent gating based on size (forward scatter, FSC) and granularity (side scatter, SSC). **Abbreviations:** PBMC: peripheral blood mononuclear cell, Tc; cytotoxic Tcell (CD3+CD8+), Th; Thelper cell (CD3+CD4+).

**Supplementary Figure 2 | Gating strategy used for the intracellular cytokine panel.** Strategy for the gating of CD4 positive T cells are given for one representative control sample (gating strategy for CD8+ T cells is identical).

**Supplementary Figure 3 | The proportions of naive and memory cells in Th (CD4^+^) and Tc (CD8^+^) cells.** Top: gating strategy used to identify naïve (T_N_, CD45RO^-^CD27^+^), memory (T_M_, CD45RO^+^) and effector T cells (T_E_, CD45RO^-^CD27^-^) within the CD3+CD4+ and CD3+CD8+ gates. Bottom: distribution of CD4 and CD8 T_E_, T_M_, T_N_ for all groups. **Abbreviations:** AU: HLA-B27 associated anterior uveitis, BU: Birdshot uveitis, HC: healthy control, IU: idiopathic intermediate uveitis, UV: Combined Uveitis samples, Tc; cytotoxic Tcell, Th; Thelper cell, T_E_; effector Tcells (CD45RO-CD27-), T_N_; naïve (CD45RO-CD27+), T_M_; memory (CD45RO+)

**Supplementary Figure 4 | Details from unsupervised automatic gating using FlowSOM.** Using FlowSOM, we clustered all individual cells within the single-cell gates of all samples into 100 distinct clusters based on the surface protein expression. Using unsupervised clustering, the 100 clusters were clustered into 22 meta-clusters of different cell types and organized in the minimal spanning tree on the right. The meta-clusters are represented by unique colors. For each cluster (i.e., cell population), pie charts indicate the relative expression for each of the different surface markers and the pie size corresponds to the number of cells in each population. Scatter plots of each cluster comparing healthy controls (blue) and uveitis patients (orange) are given for each respective meta-cluster. Clusters that differed significantly between the groups (based on Wilcoxon rank-sum test) are indicated by an asterisk (*).Relative densities of marker expression (logicle-transformed) for 11 surface markers and proportion (% in single cell gate) are given for all meta-clusters below.

**Supplementary Figure 5 | Clusters identified by FlowSOM differentiating between patients that need prolonged systemic IMT after inclusion.** Dot plots are given for 3 clusters significantly differentiating between IMT+ and IMT- patients. For each cluster (i.e., cell population), pie charts indicate the relative expression for each of the different surface markers. Cluster 84 and 100 showed relatively low CD3 expression and were not considered to represent T cell populations. Colors of the dots represent the metaclusters these clusters belong to (see also **Figure 3**). Bars in plots indicate median, error bars indicate interquartile range. P-values between HC and UV are from Wilcoxon rank-sum test.
